# CuCl/TMEDA/nor-AZADO-catalyzed aerobic oxidative acylation of amides with alcohols to produce imides†
†Electronic supplementary information (ESI) available. See DOI: 10.1039/c8sc01410h


**DOI:** 10.1039/c8sc01410h

**Published:** 2018-05-07

**Authors:** Kengo Kataoka, Keiju Wachi, Xiongjie Jin, Kosuke Suzuki, Yusuke Sasano, Yoshiharu Iwabuchi, Jun-ya Hasegawa, Noritaka Mizuno, Kazuya Yamaguchi

**Affiliations:** a Department of Applied Chemistry , School of Engineering , The University of Tokyo , 7-3-1 Hongo , Bunkyo-ku , Tokyo 113-8656 , Japan . Email: kyama@appchem.t.u-tokyo.ac.jp; b Department of Chemistry and Biotechnology , School of Engineering , The University of Tokyo , 7-3-1 Hongo , Bunkyo-ku , Tokyo 113-8656 , Japan . Email: t-jin@mail.ecc.u-tokyo.ac.jp; c Department of Organic Chemistry , Graduate School of Pharmaceutical Sciences , Tohoku University , 6-3 Aza-Aoba, Aramaki, Aoba-ku , Sendai 980-8578 , Japan; d Institute for Catalysis , Hokkaido University , Kita 21 Nishi 10 , Kita-ku , Sapporo 001-0021 , Japan

## Abstract

Although aerobic oxidative acylation of amides with alcohols would be a good complement to classical synthetic methods for imides (*e.g.*, acylation of amides with activated forms of carboxylic acids), to date, there have been no reports on oxidative acylation to produce imides. In this study, we successfully developed, for the first time, an efficient method for the synthesis of imides through aerobic oxidative acylation of amides with alcohols by employing a CuCl/TMEDA/nor-AZADO catalyst system (TMEDA = teramethylethylendiamine; nor-AZADO = 9-azanoradamantane *N*-oxyl). The proposed acylation proceeds through the following sequential reactions: aerobic oxidation of alcohols to aldehydes, nucleophilic addition of amides to the aldehydes to form hemiamidal intermediates, and aerobic oxidation of the hemiamidal intermediates to give the corresponding imides. This catalytic system utilizes O_2_ as the terminal oxidant and produces water as the sole by-product. An important point for realizing this efficient acylation system is the utilization of a TMEDA ligand, which, to the best of our knowledge, has not been employed in previously reported Cu/ligand/*N*-oxyl systems. Based on experimental evidence, we consider that plausible roles of TMEDA involve the promotion of both hemiamidal oxidation and regeneration of an active Cu^II^–OH species from a Cu^I^ species. Here promotion of hemiamidal oxidation is particularly important. Employing the proposed system, various types of structurally diverse imides could be synthesized from various combinations of alcohols and amides, and gram-scale acylation was also successful. In addition, the proposed system was further applicable to the synthesis of α-ketocarbonyl compounds (*i.e.*, α-ketoimides, α-ketoamides, and α-ketoesters) from 1,2-diols and nucleophiles (*i.e.*, amides, amines, and alcohols).

## Abstract 2

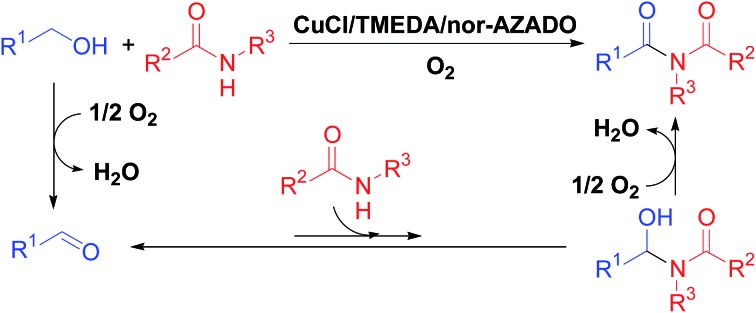
Employing a CuCl/TMEDA/nor-AZADO catalyst system, various types of structurally diverse imides could be synthesized from various combinations of alcohols and amides.

## Introduction

**Scheme 1:**
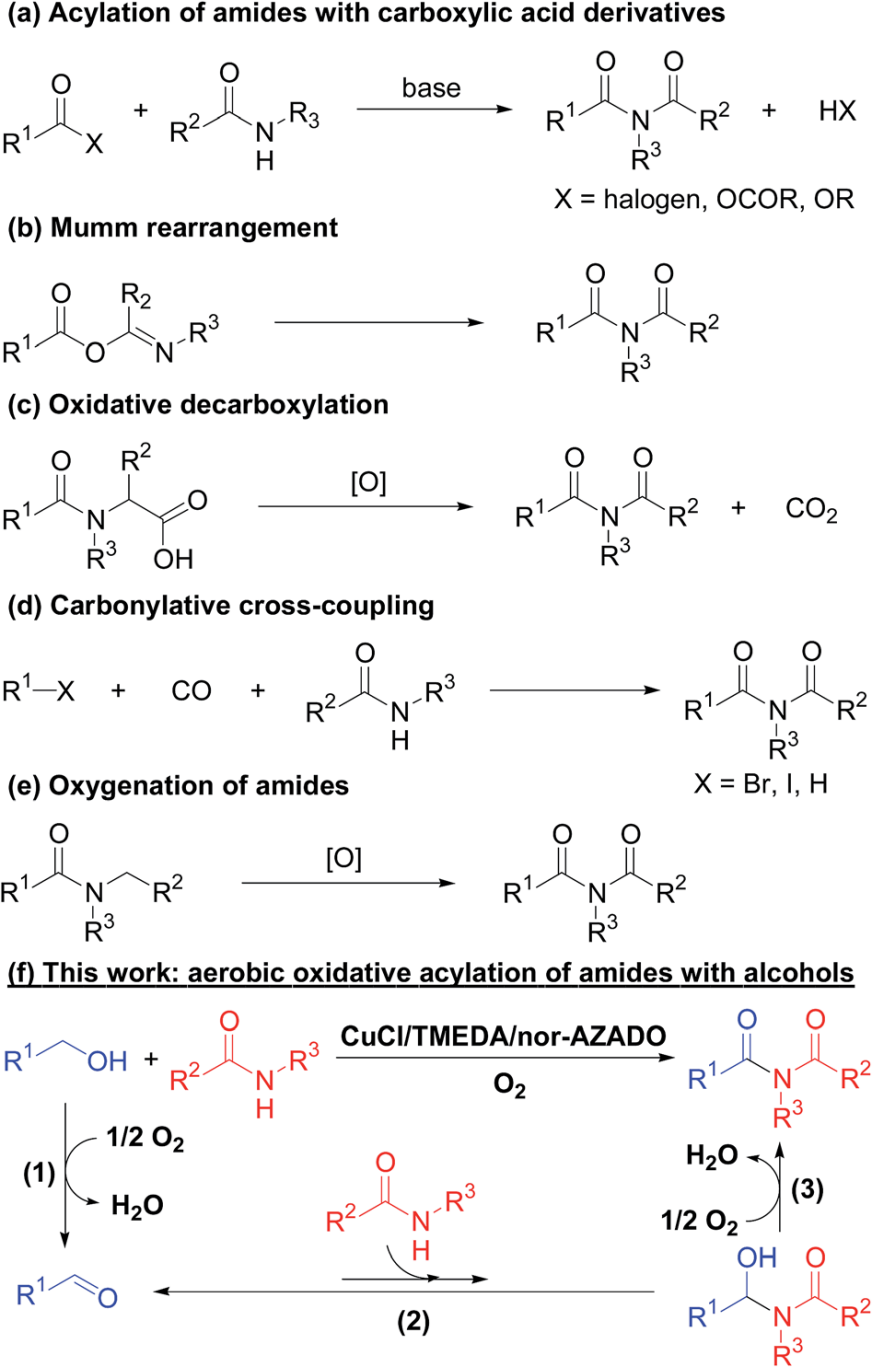
Synthetic methods for imides.

Imides are very important structural motifs found extensively in various pharmaceuticals,^1^ natural products,^2^ and industrial materials.^3^ To date, various synthetic methods for imides have been developed, including (i) acylation of amides with activated forms of carboxylic acids, such as acid chlorides, anhydrides, and esters (Scheme 1a);^4^ (ii) Mumm rearrangement of isoimides (Scheme 1b);^5^ (iii) oxidative decarboxylation of amino acids (Scheme 1c);^6^ (iv) carbonylative coupling of aryl halides and amides (Scheme 1d);^7^ and (v) oxygenation of amides (Scheme 1e).^8^ Among these methods, acylation of amides with acyl chlorides in the presence of (super)stoichiometric amounts of strong bases is the most frequently utilized. However, this antiquated acylation method has a limited substrate scope and often suffers from low synthetic and atom efficiencies due to the use of pre-functionalized substrates (*i.e.*, activated forms of carboxylic acids), which results in (super)stoichiometric amounts of wastes during both the acylation and pre-functionalization steps. Therefore, development of novel, green, and efficient methods to synthesize imides is important. In this context, aerobic oxidative acylation of amides with alcohols would provide a powerful alternative synthesis method given (i) readily available amides and alcohols as substrates without pre-functionalization; (ii) aldehydes generated *in situ* by oxidation of alcohols as the acylating reagent (using aldehydes as substrates is also possible); and (iii) O_2_ as the terminal oxidant with water as the sole by-product. However, to the best of our knowledge, there are no general and efficient methods for aerobic oxidative acylation of amides with alcohols (or even aldehydes) to produce structurally diverse imides.

Inspired by recent developments in oxidative acylation of various nucleophiles, such as alcohols or amines, with alcohols,^9^ we expected that oxidative acylation of amides with alcohols could be realized though the following sequential reactions: (1) aerobic oxidation of alcohols to aldehydes, (2) nucleophilic addition of amides to aldehydes to form hemiamidal intermediates, and (3) aerobic oxidation of the hemiamidal intermediates to give the corresponding imide products (Scheme 1f). Because step (2) is in equilibrium and the equilibrium constant is possibly small, the key to achieving the target reaction would be the development of an efficient oxidation catalyst, particularly for step (3), to drive the reaction toward the imide products. In this study, to the best of our knowledge, we have successfully developed the first powerful and practical method for synthesis of various types of structurally diverse imides through oxidative acylation of amides with alcohols using O_2_ as the terminal oxidant by employing a CuCl/TMEDA/nor-AZADO catalyst system (TMEDA = teramethylethylendiamine; nor-AZADO = 9-azanoradamantane *N*-oxyl) (Scheme 1f).

To date, numerous efficient Cu/ligand/*N*-oxyl catalyst systems have been developed for aerobic oxidation of alcohols or amines.^10^ However, only one previous study has reported Cu/ligand/*N*-oxyl-catalyzed oxidative acylation of nucleophiles with alcohols; Stahl and co-workers quite recently reported an elegant Cu/ligand/ABNO catalyst system (ABNO = 9-azabicyclo[3.3.1]nonane *N*-oxyl) for aerobic oxidative acylation of amines with alcohols to produce amides.^11^ In this study, we found, for the first time, that CuCl combined with TMEDA and nor-AZADO was a highly generalizable and practical catalyst system to convert a wide variety of alcohols and amides to produce the desired imides without tedious catalyst re-optimization even with different alcohol/amide combinations. In addition, the proposed system was further applicable to the synthesis of α-ketocarbonyl compounds from 1,2-diols and nucleophiles, such as amides, amines, and alcohols. Notably, a readily available TMEDA ligand, which, to the best of our knowledge, has never been employed as the ligand in previously reported Cu/ligand/*N*-oxyl oxidation catalyst systems, showed significantly superior performance compared to commonly utilized pyridyl-based ligands, including bipyridines and phenanthrolines. Based on experimental evidence, we concluded that plausible roles of TMEDA involve the promotion of both hemiamidal oxidation and regeneration of an active Cu^II^–OH species by oxidation of a Cu^I^ species using O_2_. Moreover, we found that nor-AZADO,^12^ developed by Iwabuchi and co-workers, was more efficient for the proposed acylation than other generally utilized *N*-oxyls, such as TEMPO,^13^ ABNO,^14^ and AZADO^15^ (TEMPO = 2,2,6,6-tetramethylpiperidine *N*-oxyl; AZADO = 2-azaadamantane *N*-oxyl).

## Results and discussion

### Optimization of reaction conditions

Initially, we carried out oxidative acylation of 2-pyrrolidone (**2a**) with benzyl alcohol (**1a**) using a CuCl/TMEDA/nor-AZADO catalyst system under 1 atm of O_2_ at room temperature (*ca.* 23 °C) in tetrahydrofuran (THF). Under the conditions described in Table 1 without molecular sieves 3A (MS 3A), the desired 1-benzoyl-2-pyrrolidone (**3aa**) was obtained in 15% yield with a 75% yield of benzaldehyde (**4a**) derived from the oxidation of **1a** (Table 1, entry 1). To promote reactions effectively, activated (dried) MS 3A was utilized frequently to remove water formed during the reactions in previously reported Cu/ligand/*N*-oxyl catalyst systems.^10^,^11^ In the proposed system, the effect of activated MS 3A was also very significant; when activated MS 3A was added to the reaction mixture in advance, **3aa** was obtained in quantitative yield within only 5 min (Table 1, entry 2). However, the reaction did not efficiently proceed in the presence of non-activated MS 3A (containing *ca.* 20 wt% water) (Table 1, entry 3). Thus, water formed during the reaction likely inhibits the reaction, and the use of activated (dried) MS 3A is very important to promote the reaction effectively. During the catalytic turnover, the reaction solution was almost colorless. When **1a** was completely converted into **3aa** (after 5 min, in the case of Table 1, entry 2), the reaction solution became light green.

**Table 1:** Optimization of the reaction conditions^*a*^

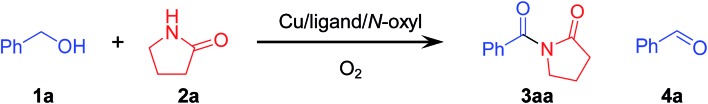							
Entry	Cu source	Ligand	*N*-Oxyl	Time (min)	Conv. or yield (%)		
					**1a** (%)	**3aa**	**4a**
1^*b*^	CuCl	TMEDA	nor-AZADO	5	>99	15	75
**2**	**CuCl**	**TMEDA**	**nor-AZADO**	**5**	**>99**	**>99**	**<1**
3^*c*^	CuCl	TMEDA	nor-AZADO	5	>99	10	80
4	CuCl	bpy	nor-AZADO	5	>99	10	83
5	CuCl	^4Me^bpy	nor-AZADO	5	99	2	87
6	CuCl	^6Me^bpy	nor-AZADO	5	70	<1	60
7	CuCl	^4MeO^bpy	nor-AZADO	5	>99	14	78
8	CuCl	phen	nor-AZADO	5	8	<1	1
9	CuCl	^MeO^phen	nor-AZADO	5	7	<1	2
10	CuCl	TPA	nor-AZADO	5	38	<1	28
11	CuCl	TEEDA	nor-AZADO	5	>99	21	72
12	CuCl	TMEDA	nor-AZADO	3	>99	61	38
13	CuCl	TMEDA	TEMPO	3	11	<1	5
14	CuCl	TMEDA	ABNO	3	>99	31	66
15	CuCl	TMEDA	keto-ABNO	3	90	41	45
16	CuCl	TMEDA	AZADO	3	>99	31	62
17	CuCl	TMEDA	1-Me-AZADO	3	>99	49	54
18	CuCl	TMEDA	5-F-AZADO	3	99	37	55
19	CuCl	TMEDA	4-Cl-AZADO	3	97	49	46
20	CuBr	TMEDA	nor-AZADO	5	>99	69	30
21	CuI	TMEDA	nor-AZADO	5	15	<1	6
22	CuOAc	TMEDA	nor-AZADO	5	66	2	52
23	CuCl_2_	TMEDA	nor-AZADO	5	28	<1	22
24	—	TMEDA	nor-AZADO	5	6	<1	1
25	CuCl	—	nor-AZADO	5	30	<1	22
26	CuCl	TMEDA	—	5	7	<1	1
27^*d*^	CuCl	TMEDA	nor-AZADO	5	10	<1	1
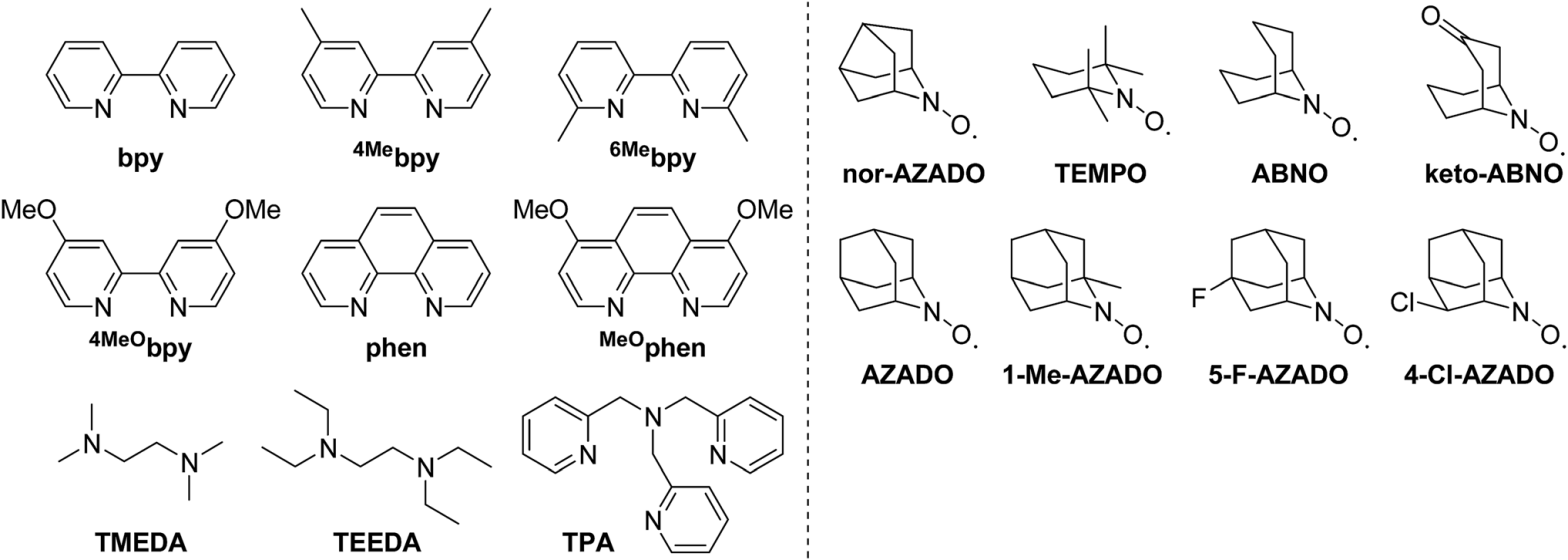							

^*a*^Reaction conditions: **1a** (0.5 mmol), **2a** (0.55 mmol), Cu source (5 mol%), ligand (5 mol%), *N*-oxyl (3 mol%), activated MS 3A (200 mg), THF (2.5 mL), O_2_ (balloon), room temp. (*ca.* 23 °C). Conversion and yields were determined by GC analysis.

^*b*^Without MS 3A.

^*c*^Using non-activated MS 3A (containing *ca.* 20 wt% water).

^*d*^Ar (1 atm).

Among the ligands examined, TMEDA was the best ligand for acylation of **2a** with **1a** (Table 1, entry 2). We confirmed by careful GC and GC-MS analyses that the mass balance for TMEDA was almost preserved and by-products derived from TMEDA were not detected at all after the reaction, thus indicating that oxidative decomposition of TMEDA hardly occurred under the present reaction conditions. Other commonly utilized ligands in previously reported Cu/ligand/*N*-oxyl catalyst systems for several oxidation reactions, such as bpy (2,2′-bipyridyl), ^4Me^bpy (4,4′-dimethyl-2,2′-bipyridyl), ^6Me^bpy (6,6′-dimethyl-2,2′-bipyridyl), ^4MeO^bpy (4,4′-dimethoxy-2,2′-bipyridyl), phen (1,10-phenanthroline), ^MeO^phen (4,7-dimethoxy-1,10-phenanthroline), and TPA (tris(2-pyridylmethyl)amine), were all inferior to TMEDA under the same reaction conditions (Table 1, entries 4–10). Another aliphatic *N*-based bidentate ligand, *i.e.*, TEEDA (*N*,*N*,*N*′,*N*′-tetraethylethylenediamine), also promoted the reaction but was less effective than TMEDA (Table 1, entry 11). These results indicate that ligands play a crucial role in the present reaction. Peralkylated diamine ligands, particularly TMEDA, could effectively facilitate the reaction. Thus, TMEDA was the key to efficient acylation of amides with alcohols. The roles of TMEDA in the proposed catalyst system are discussed in detail in a later section.

Various *N*-oxyls, such as nor-AZADO, TEMPO, ABNO, keto-ABNO (9-azabicyclo[3.3.1]nonane-3-one *N*-oxyl), AZADO, 1-Me-AZADO (1-methyl-2-azaadamantane-*N*-oxyl), 5-F-AZADO (5-fluoro-2-azaadamantane-*N*-oxyl), and 4-Cl-AZADO (4-chloro-2-azaadamantane-*N*-oxyl), were examined for the acylation of **2a** with **1a**. Among these *N*-oxyls, nor-AZADO was the most effective for the acylation (Table 1, entry 12). In the presence of the other *N*-oxyls (except for TEMPO), the reaction also proceeded accordingly (Table 1, entries 13–19). Other Cu sources, such as CuBr, CuI, CuOAc, and CuCl_2_, were much less effective than CuCl (Table 1, entry 2 *vs.* entries 20–23).^16^ Several control experiments revealed that acylation did not proceed at all when lacking any one of CuCl, TMEDA, or nor-AZADO (Table 1, entries 24–26). In addition, **3aa** was hardly obtained under 1 atm of Ar, which indicates that O_2_ functioned as the terminal oxidant (Table 1, entry 27). THF was the best solvent for the proposed acylation system. We confirmed that THF used in this study did not contain any peroxides before the reaction. In addition, after the reaction, no peroxides were also detected in the reaction solution. Moreover, the reaction also efficiently proceeded using THF containing an antioxidant 2,6-di-*tert*-butyl-*p*-cresol. When the solvent was changed from THF to dichloromethane (DCM) or acetonitrile (MeCN), the yield of **3aa** decreased drastically (Table S1, ESI†).

### Substrate scope for imide synthesis

With the optimized reaction conditions in hand, we examined the substrate scope for the proposed CuCl/TMEDA/nor-AZADO-catalyzed oxidative acylation of amides with alcohols. Various kinds of structurally diverse imides could be synthesized starting from various combinations of alcohols and amides (Tables 2 and 3). The substrates used in this study are summarized in Fig. S1, ESI.†

**Table 2:** Substrate scope for acylation of lactams with alcohols^*a*^

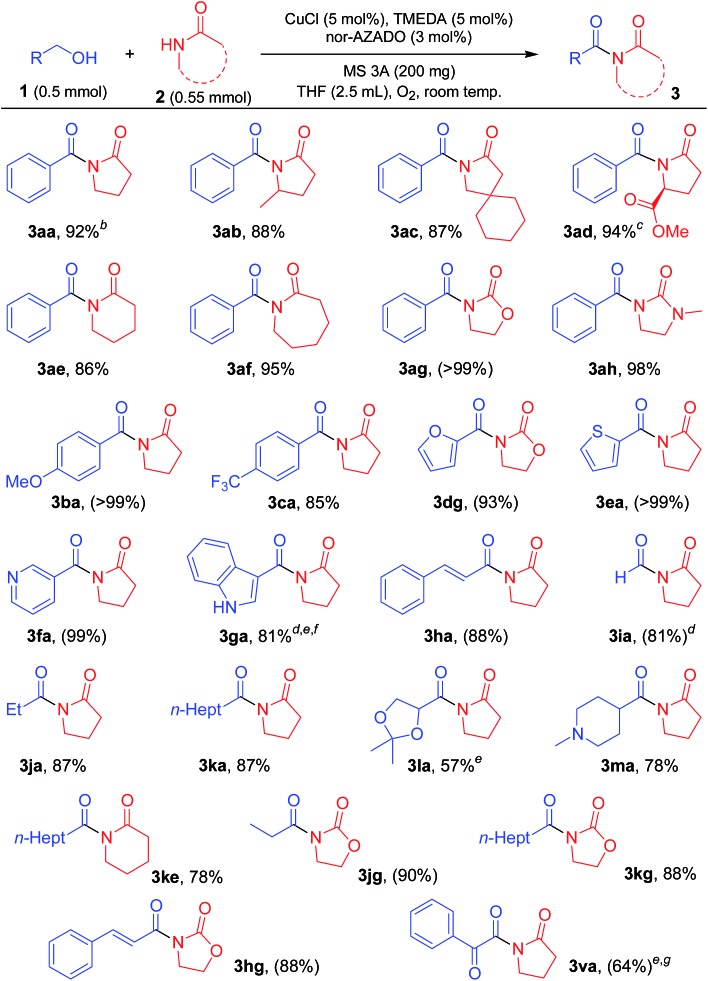

^*a*^Reaction conditions: 1 (0.5 mmol), 2 (0.55 mmol), CuCl (5 mol%), TMEDA (5 mol%), nor-AZADO (3 mol%), MS 3A (200 mg), THF (2.5 mL), O_2_ (balloon), room temp. (*ca.* 23 °C), 1 h. Isolated yields are shown. Values in parentheses are GC yields.

^*b*^5 min.

^*c*^>99% ee.

^*d*^24 h.

^*e*^2 (1.0 mmol).

^*f*^CuCl (10 mol%), TMEDA (10 mol%), nor-AZADO (6 mol%).

^*g*^K_2_CO_3_ (20 mol%).

**Table 3:** Substrate scope for acylation of primary amides with alcohols^*a*^

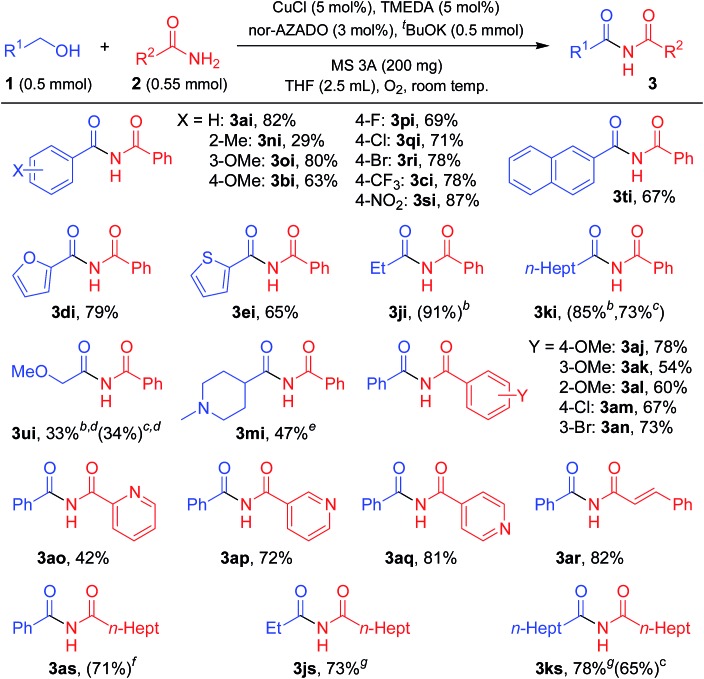

^*a*^Reaction conditions: 1 (0.5 mmol), 2 (0.55 mmol), CuCl (5 mol%), TMEDA (5 mol%), nor-AZADO (3 mol%), ^*t*^BuOK (0.5 mmol), MS 3A (200 mg), THF (2.5 mL), O_2_ (balloon), room temp. (*ca.* 23 °C), 24 h. Isolated yields are shown. GC yields are shown in parentheses.

^*b*^4-Cl-AZADO (3 mol%), without ^*t*^BuOK.

^*c*^Without ^*t*^BuOK.

^*d*^ **2i** (1.0 mmol).

^*e*^CuCl (10 mol%), TMEDA (10 mol%), nor-AZADO (6 mol%), without ^*t*^BuOK.

^*f*^ ^*t*^BuOK (0.25 mmol).

^*g*^1-Me-AZADO (3 mol%), without ^*t*^BuOK.

As shown in Table 2, various lactams could be acylated to produce the corresponding imides in moderate to high yields. Various substituents, including an ester group on 5-membered lactams, reacted efficiently with **1a** to afford the corresponding imides in high yields (Table 2, **3aa**, **3ab**, **3ac**, and **3ad**). When an enantiomerically pure lactam was used as the substrate, the corresponding imide was obtained in high yield with retention of the stereochemistry (>99% ee) (Table 2, **3ad**). In addition, 6- or 7-membered lactams were good substrates for the present reaction, affording the corresponding imides in high yields (Table 2, **3ae** and **3af**). Heteroatom-substituted lactams, such as 2-oxazolidone and 1-methyl-2-imidazolidone, were also good substrates, and acylation with various alcohols gave the corresponding imides in excellent yields (Table 2, **3ag**, **3ah**, **3dg**, **3jg**, **3kg**, and **3hg**). Benzylic alcohols with electron-donating or withdrawing substituents were all good partners for **2a** (Table 2, **3ba** and **3ca**). The acylation proceeded smoothly with various alcohols containing heteroaromatic rings, such as furan, thiophene, pyridine, and indole rings (Table 2, **3dg**, **3ea**, **3fa**, and **3ga**). An α,β-unsaturated alcohol, *i.e.*, cinnamyl alcohol, also reacted efficiently with amides, affording the corresponding imides without oxidation of the double bond (Table 2, **3ha** and **3hg**). The reaction proceeded well even when aliphatic alcohols were used as acylating reagents (Table 2, **3ia**, **3ja**, **3ka**, **3la**, **3ma**, **3ke**, **3jg**, and **3kg**). An acetal group was also tolerated in the present reaction (Table 2, **3la**). Note that methanol could be utilized as an efficient formylation reagent for **2a** (Table 2, **3ia**). Interestingly, when the reaction of styrene glycol (1,2-diol) and **2a** was performed, both the primary and secondary alcohol groups in styrene glycol were smoothly oxidized to form the corresponding α-ketoaldehyde, followed by the reaction with **2a** to afford the corresponding α-ketoimide (Table 2, **3va**).

Next, the scope for the acylation of primary amides was examined. The optimized conditions for lactams turned out to be inefficient for acylation of benzamide (**2i**) with **1a**, and only a stoichiometric amount of the corresponding imide (**3ai**) with respect to Cu was obtained (Table S2, entries 1–3, ESI†). Acidic groups, *e.g.*, carboxylic acids and phenols, have been shown to poison Cu/ligand/*N*-oxyl-catalyzed alcohol oxidation, possibly by inhibiting formation of the requisite Cu-alkoxide species. The secondary imide has a p*K*_a_ value similar to that of a carboxylic acid, and thus we consider that strong bases can ensure that the imide is deprotonated and unable to inhibit catalytic turnover. Indeed, the reaction proceeded efficiently in the presence of potassium *tert*-butoxide (^*t*^BuOK), giving **3ai** in quantitative yield (Table S2, entry 5, ESI†). Among the bases examined, ^*t*^BuOK was the best base additive (Table S2, entries 5 and 22–24, ESI†). Thus, we utilized ^*t*^BuOK for acylation of primary amides as required.

As shown in Table 3, various primary amides were acylated efficiently to give the corresponding imides. Benzylic alcohols with electron-donating and electron-withdrawing substituents on the aromatic rings and 2-naphthalenemethanol successfully reacted with **2i** to afford the corresponding imides (Table 3, **3ai**, **3ni**, **3oi**, **3bi**, **3pi**, **3qi**, **3ri**, **3ci**, **3si**, and **3ti**). When using 2-methylbenzyl alcohol for acylation of **2i**, the yield of the corresponding imide was not satisfactory, likely due to the steric hinderance of the 2-methyl group (Table 3, **3ni**). Notably, with halo-substituted benzylic alcohols, the desired imides were obtained without dehalogenation, which enables further functionalization of these imide products (Table 3, **3pi**, **3qi**, and **3ri**). Alcohols containing heteroaromatic rings, such as furan and thiophene rings, reacted efficiently with **2i** (Table 3, **3di**, and **3ei**). Aliphatic alcohols also reacted smoothly with **2i** (Table 3, **3ji**, and **3ki**). However, heteroatoms containing aliphatic alcohols were somewhat less effective acylation sources (Table 3, **3ui**, and **3mi**). Benzamides with various substituents on 2-, 3- or 4-positions were all similarly effective for acylation with **1a** (Table 3, **3aj**, **3ak**, **3al**, **3am**, and **3an**). Halo-substituted benzamides were also acylated without dehalogenation (Table 3, **3am** and **3an**). In addition, 3- and 4-picolinamides were efficiently converted into the corresponding imides (Table 3, **3ap**, and **3aq**), while 2-picolinamide was less effective, possibly due to its bidentate coordination to the active Cu center (Table 3, **3ao**). An α,β-unsaturated amide, *i.e.*, cinnamamide, also reacted efficiently with **1a** (Table 3, **3ar**). An aliphatic amide could also be acylated with various alcohols, including benzylic and aliphatic alcohols, to give the corresponding imides (Table 3, **3as**, **3js**, and **3ks**).

**Scheme 2:**
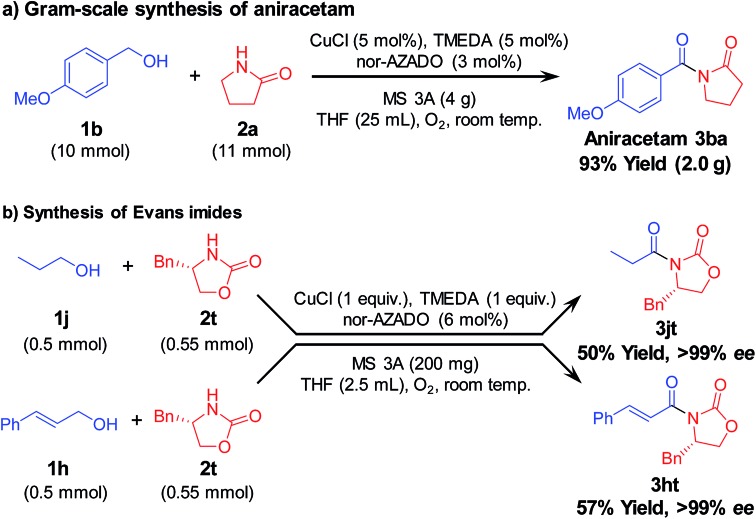
Gram-scale aniracetam synthesis and Evans imide synthesis. Reaction conditions for (a) **1b** (10 mmol), **2a** (11 mmol), CuCl (5 mol%), TMEDA (5 mol%), nor-AZADO (3 mol%), MS 3A (4 g), THF (25 mL), O_2_ (balloon), room temp. (*ca.* 23 °C), 2 h; for (b) 1 (0.5 mmol), 2 (0.55 mmol), CuCl (1 eq.), TMEDA (1 eq.), nor-AZADO (6 mol%), MS 3A (200 mg), THF (2.5 mL), O_2_ (balloon), room temp. (*ca.* 23 °C), 24 h. Isolated yields are shown.

To further demonstrate the practical applicability of the proposed CuCl/TMEDA/nor-AZADO-catalyzed oxidative acylation system to produce imides, we performed gram-scale (10 mmol-scale) synthesis of “aniracetam” (**3ba**), which is a class of racetams sold as an anxiolytic drug.1*a* When 4-methoxybenzyl alcohol (**1b**) and **2a** were used as starting materials, the reaction proceeded efficiently to obtain 2.0 g of **3ba** (93% isolated yield) (Scheme 2a). Next, using the proposed acylation method, we attempted to synthesize chiral *N*-acyloxazolidinones (known as “Evans imides”), which are widely used as starting materials for various enantio-selective syntheses, such as alkylation, aldol reaction, and Michael addition.^17^ Although stoichiometric amounts of CuCl and TMEDA were required, the acylation of a chiral oxazolidinone, *i.e.*, (*S*)-4-benzyl-2-oxazolidinone (**2t**), with 1-propanol (**1j**) or cinnamyl alcohol (**1h**) gave the corresponding Evans imides in moderate yields with >99% ee (Scheme 2b).

### Reaction mechanism and roles of TMEDA

**Scheme 3:**
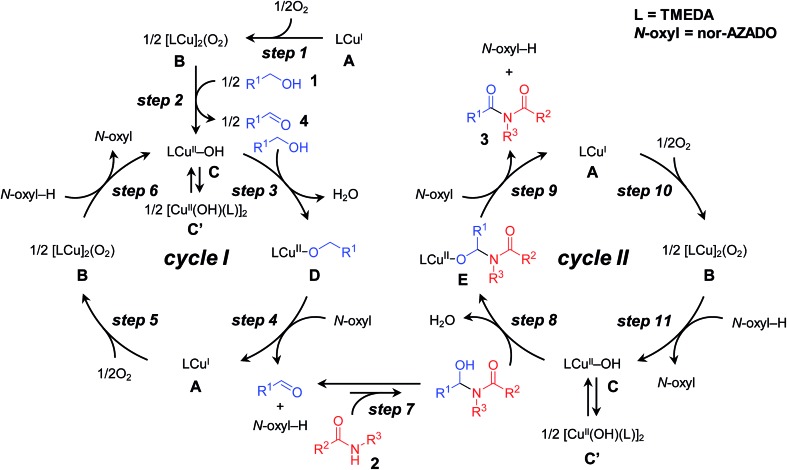
Plausible reaction mechanism for proposed CuCl/TMEDA/nor-AZADO-catalyzed oxidative acylation of amides with alcohols.

From the reaction profile for the CuCl/TMEDA/nor-AZADO-catalyzed oxidative acylation of 2-pyrrolidone (**2a**) with benzyl alcohol (**1a**) to produce the corresponding imide **3aa**, it is clear that the present reaction proceeds *via* benzaldehyde (**4a**) as the intermediate (Scheme 1f, Table 1). In a separate experiment, we confirmed that **3aa** was obtained quantitatively starting from **4a** and **2a** (Table S3, entry 1, ESI†). A plausible reaction mechanism for the proposed CuCl/TMEDA/nor-AZADO-catalyzed acylation of amides with alcohols is shown in Scheme 3.^18^ Initially, Cu^I^ species **A** is oxidized by O_2_ to generate dicopper–oxygen complex **B**, which is then reduced by an alcohol to afford active Cu^II^–OH species **C** (Scheme 3, steps 1 and 2).^19^ Species **C** may be in equilibrium with Cu^II^_2_ bis-μ-hydroxo dimer **C′**.18b,^20^ Then, Cu^II^-alkoxide species **D** is formed by ligand exchange between species **C** and an alcohol with the liberation of water (Scheme 3, step 3). In the presence of nor-AZADO (*N*-oxyl), an aldehyde intermediate was formed from species **D** concomitant with its reduction to Cu^I^ species **A** and the formation of the corresponding *N*-hydroxylamine (*N*-oxyl-H) (Scheme 3, step 4). Species **A** is then re-oxidized by O_2_ to afford species **B**, followed by its reduction by *N*-oxyl-H (or an alcohol) to regenerate active Cu^II^–OH species **C** to complete the catalytic cycle I (Scheme 3, steps 5 and 6). Next, a hemiamidal intermediate is formed by nucleophilic addition of an amide to the aldehyde generated in the catalytic cycle I (Scheme 3, step 7).^21^ The oxidation of the hemiamidal intermediate by a catalytic cycle as with cycle I gives the final imide product (Scheme 3, cycle II).

**Fig. 1:**
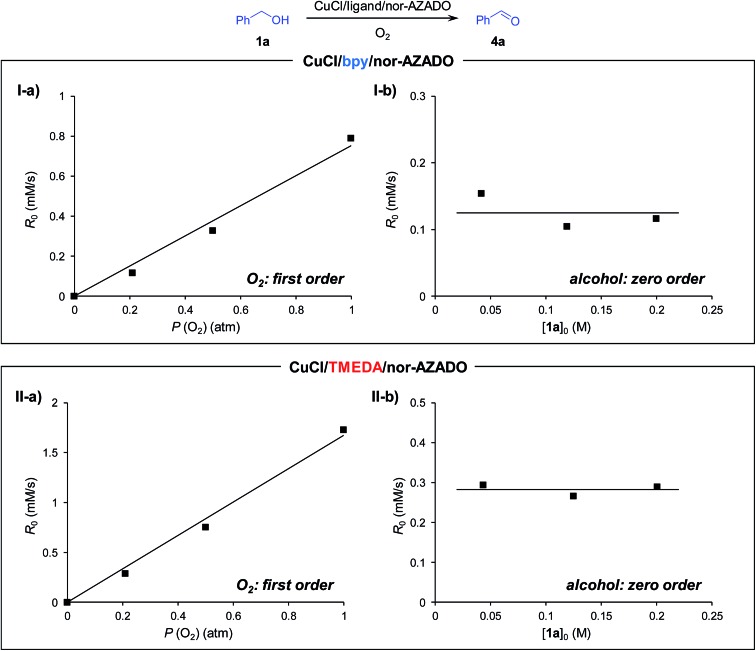
Dependence of (I) CuCl/bpy/nor-AZADO-catalyzed and (II) CuCl/TMEDA/nor-AZADO-catalyzed alcohol oxidation on (a) the partial pressure of O_2_ and (b) concentration of **1a**. Line fit: *R*_0_ = 0.754*P* (O_2_) (I-a, *R*^2^ = 0.985), *R*_0_ = 1.672*P* (O_2_) (II-a, *R*^2^ = 0.992). Reaction conditions for (a): **1a** (0.2 M), CuCl (10 mM), ligand (10 mM), nor-AZADO (6 mM), MS 3A (200 mg), THF (2.5 mL), O_2_ (0.2–1.0 atm), room temp. (*ca.* 23 °C); for (b): **1a** (0.04–0.20 M), CuCl (10 mM), ligand (10 mM), nor-AZADO (6 mM), MS 3A (200 mg), THF (2.5 mL), O_2_ (0.2 atm), room temp. (*ca.* 23 °C). Yields were determined by GC analysis. Raw data are summarized in Fig. S2, ESI.†

**Scheme 4:**
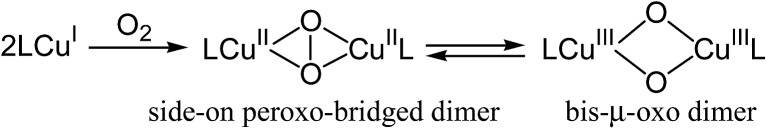
Reaction of a Cu^I^ species and O_2_ in the presence of suitable ligands.

Recently, Stahl *et al.* proposed the catalytic cycle I for Cu/ligand/*N*-oxyl-catalyzed aerobic alcohol oxidation based on careful kinetic, spectroscopic, and quantum chemical studies.^18^,^22^ We consider that the CuCl/TMEDA/nor-AZADO-catalyzed aerobic alcohol oxidation also proceeds through cycle I. Kinetic studies for CuCl/TMEDA/nor-AZADO-catalyzed oxidation of **1a** to **4a** revealed that the reaction rate showed first-order dependence on O_2_ pressure (Fig. 1(II-a)) and was nearly independent of the **1a** concentration (Fig. 1(II-b)), thereby indicating that the regeneration of an active Cu^II^–OH species from a Cu^I^ species (Scheme 3, step 5 or 6) is included in the rate-limiting step. To reveal the effect of TMEDA, similar kinetic studies were performed using a commonly utilized bpy ligand. When using bpy, the reaction rate exhibited first-order dependence on O_2_ pressure (Fig. 1(I-a)) and zero-order dependence on the **1a** concentration (Fig. 1(I-b)). Therefore, the Cu^II^–OH regeneration is also included in the rate-limiting step in the case of bpy. From these kinetic studies, we revealed that the rate for Cu^II^–OH regeneration using TMEDA was *ca.* twice that of using bpy (Fig. 1). It is known that the reaction of Cu^I^ species and O_2_ gives various copper–oxygen species and that the reaction in the presence of suitable ligands yield an equilibrium mixture of a CuII2 side-on peroxo-bridged dimer and a CuIII2 bis-μ-oxo dimer (Scheme 4).^23^ To date, many reports have considered the formation and reactivity of the bis-μ-oxo dimer, and the following points have been discussed:^24^ (i) the bis-μ-oxo dimer is a better hydrogen-acceptor compared to the side-on peroxo-bridged dimer, (ii) peralkylated diamine ligands can promote formation of the bis-μ-oxo dimer, and (iii) the bis-μ-oxo dimer prepared using TMEDA is relatively stable, and its limited steric demands allow efficient oxidation (hydrogen abstraction) of exogenous substrates (*e.g.*, benzyl alcohol).^19^ We consider that the promotional effect of TMEDA for Cu^II^–OH regeneration is likely due to the above-mentioned three points, *i.e.*, increasing the concentration of the bis-μ-oxo dimer (Scheme 3, step 5) and/or promoting hydrogen abstraction from *N*-oxyl-H (or an alcohol) (Scheme 3, step 6).

**Scheme 5:**
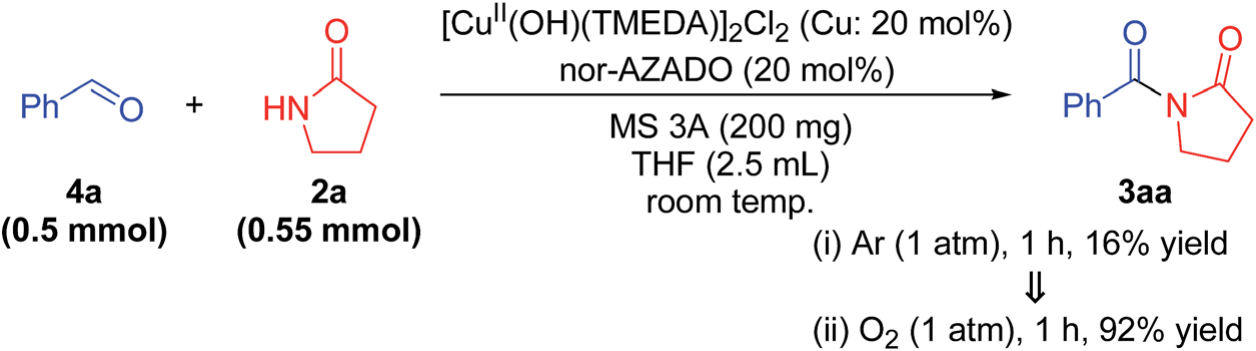
Control experiments.

Although TMEDA effectively promotes Cu^II^–OH regeneration, the big difference between acylation using TMEDA and that using bpy (Table 1, entry 2 *vs.* entry 3) cannot be explained only by the aforementioned promotional effect. Consequently, we consider that the effect of TMEDA should be most obvious on the hemiamidal oxidation step in cycle II (possibly Scheme 3, step 9). Indeed, when acylation of **2a** with **4a** was carried out under the conditions described in Table S3, ESI,† the yields of **3aa** using TMEDA and bpy were 98% and 17%, respectively (Table S3, entries 1 and 2, ESI†).^25^ In addition, the production rate (initial rate) of **3aa** using TMEDA was at least *ca.* eight times greater than that using bpy. Therefore, we investigated the effect of TMEDA on the hemiamidal oxidation step in more detail. As mentioned previously, we consider that the hemiamidal oxidation likely proceeds through the catalytic cycle II. To reveal this, the following control experiments were conducted. When the acylation of **2a** with **4a** using [Cu(OH)(TMEDA)]_2_Cl_2_ (Cu: 20 mol% with respect to **4a**) instead of CuCl under non-aerobic conditions (1 atm of Ar), a nearly stoichiometric amount of **3aa** (16% yield base on **4a**) with respect to the Cu^II^ species used was produced, and then the reaction stopped (Scheme 5). This result is in accord with the left half of cycle II in Scheme 3, *i.e.*, once the Cu^II^–OH species **C** and *N*-oxyl are reduced to Cu^I^ species **A** and *N*-oxyl-H, respectively, the catalytic turnover stops in the absence of O_2_. In addition, the monomeric Cu^II^–OH species **C** and CuII2 bis-μ-hydroxo dimer **C′** are likely in equilibrium. After this non-aerobic reaction, O_2_ was introduced to the reactor, and the reaction was continued successively. In this case, the yield of **3aa** increased up to 92% (Scheme 5). Therefore, O_2_ can act as the oxidant to re-oxidize Cu^I^ species **A** to regenerate the active Cu^II^–OH species **C** (the right half of cycle II in Scheme 3), thereby leading to complete conversion of the substrates into the desired imide product.

**Fig. 2:**
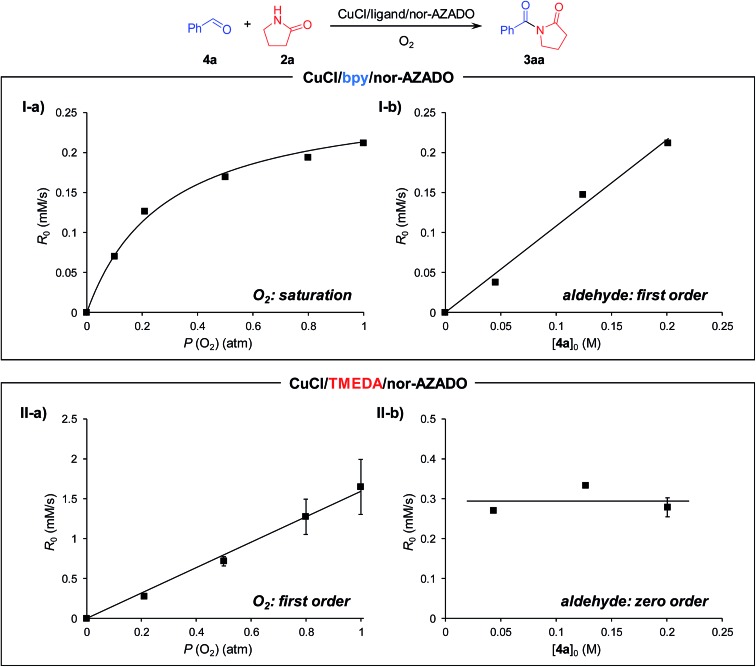
Dependence of (I) CuCl/bpy/nor-AZADO-catalyzed and (II) CuCl/TMEDA/nor-AZADO-catalyzed hemiamidal oxidation on (a) the partial pressure of O_2_ and (b) the concentration of **4a**. Line fit: *R*_0_ = 1.078[**4a**]_0_ (I-b, *R*^2^ = 0.989), *R*_0_ = 1.594*P* (O_2_) (II-a, *R*^2^ = 0.993). Reaction conditions for (a) **4a** (0.2 M), **2a** (0.22 M), CuCl (10 mM), ligand (10 mM), nor-AZADO (6 mM), MS 3A (200 mg), THF (2.5 mL), O_2_ (0.1–1.0 atm), room temp. (*ca.* 23 °C); for (b) **4a** (0.04–0.20 M), **2a** (0.22 M), CuCl (10 mM), ligand (10 mM), nor-AZADO (6 mM), MS 3A (200 mg), THF (2.5 mL), O_2_ (0.2 or 1.0 atm), room temp. (*ca.* 23 °C). Yields were determined by GC analysis. Raw data are summarized in Fig. S3, ESI.†

Next, kinetic studies for the acylation of **2a** with **4a** to produce **3aa** were performed. When using bpy, the production rate (initial rate) of **3aa** showed saturation kinetics for O_2_ pressure (Fig. 2(I-a)) and first-order dependence on the **4a** concentration (Fig. 2(I-b)). The kinetic behavior for acylation using bpy was quite different compared to that of the alcohol oxidation. We consider that the hemiamidal oxidation (Scheme 3, step 9) is included in the rate-limiting step for the acylation of **2a** with **4a** using bpy under standard conditions (1 atm of O_2_). Therefore, with the CuCl/bpy/nor-AZADO catalyst system, hemiamidal oxidation is significantly more difficult than alcohol oxidation. Thus, the rates when using bpy are in the following order: alcohol oxidation > Cu^II^–OH regeneration > hemiamidal oxidation. Note that the kinetic behavior for acylation using TMEDA was quite different from that using bpy. The reaction rate for the CuCl/TMEDA/nor-AZADO-catalyzed acylation exhibited first-order dependence on O_2_ pressure (Fig. 2(II-a)) and zero-order dependence on the **4a** concentration (Fig. 2(II-b)). In addition, the reaction rate for acylation of **2a** with **4a** was nearly the same as that for the oxidation of **1a** to **4a** (Fig. 2(II-a and II-b) *vs.*Fig. 1(II-a and II-b)). These results strongly indicate that Cu^II^–OH regeneration (Scheme 3, step 10 or 11) is included in the rate-limiting step for the CuCl/TMEDA/nor-AZADO-catalyzed hemiamidal oxidation (acylation of **2a** with **4a**), as with the alcohol oxidation (oxidation of **1a**) (*vide supra*). Therefore, with TMEDA, both the alcohol oxidation and hemiamidal oxidation are significantly faster than the Cu^II^–OH regeneration.

From these kinetic results, we concluded that, in the proposed CuCl/TMEDA/nor-AZADO-catalyzed acylation of amides with alcohols, TMEDA promotes both hemiamidal oxidation (Scheme 3, step 9) and Cu^II^–OH regeneration (Scheme 3, step 5 or 6; Scheme 3, step 10 or 11), and that the effect of TMEDA on the hemiamidal oxidation is very significant. In the proposed CuCl/TMEDA/nor-AZADO-catalyzed acylation of amides with alcohols, Cu^II^–OH regeneration (Scheme 3, step 5 or 6, and Scheme 3, step 10 or 11) is the slower step compared to alcohol and hemiamidal oxidation, thereby suggesting that Cu^I^ species **A** is in the resting state. This is consistent with the above-mentioned color observation of the reaction solution, *i.e.*, the reaction solution was almost colorless during the catalytic turnover and became light green after complete conversion of the substrates into the corresponding imides.

### Synthesis of α-ketoamides and α-ketoesters

As above-mentioned, the proposed CuCl/TMEDA/nor-AZADO catalyst system was highly effective for the oxidation of styrene glycol (1,2-diol) to the corresponding α-ketoaldehyde; when the reaction of styrene glycol and amide **2a** was carried out, the corresponding α-ketoimide was obtained (Table 2, **3va**). We envisaged that this method utilizing 1,2-diols could be further applied to the synthesis of various α-ketocarbonyl compounds. From the mechanistic studies as described above, we chose a [Cu(OH)(TMEDA)]_2_Cl_2_/nor-AZADO catalyst system for the studies of α-ketocarbonyl synthesis. We found that secondary amines (*e.g.*, piperidine and *N*-methylbenzylamine) and aniline could be utilized as the nucleophiles; in the presence of a small excess of these amines, the reaction of styrene glycol gave their corresponding α-ketoamides in high yields (Table 4, upper). Unfortunately, the use of primary aliphatic amines was unsuccessful. Moreover, various kinds of alcohols, including aliphatic primary, aliphatic secondary, and benzylic ones, could also be utilized as the nucleophiles for the synthesis of their corresponding α-ketoesters (Table 4, lower). To the best of our knowledge, to date, there are no reports on such α-ketocarbonyl synthesis methods using 1,2-diols and nucleophilic reagents.

**Table 4:** Synthesis of α-ketoamides or α-ketoesters from 1,2-diols and nucleophiles^*a*^

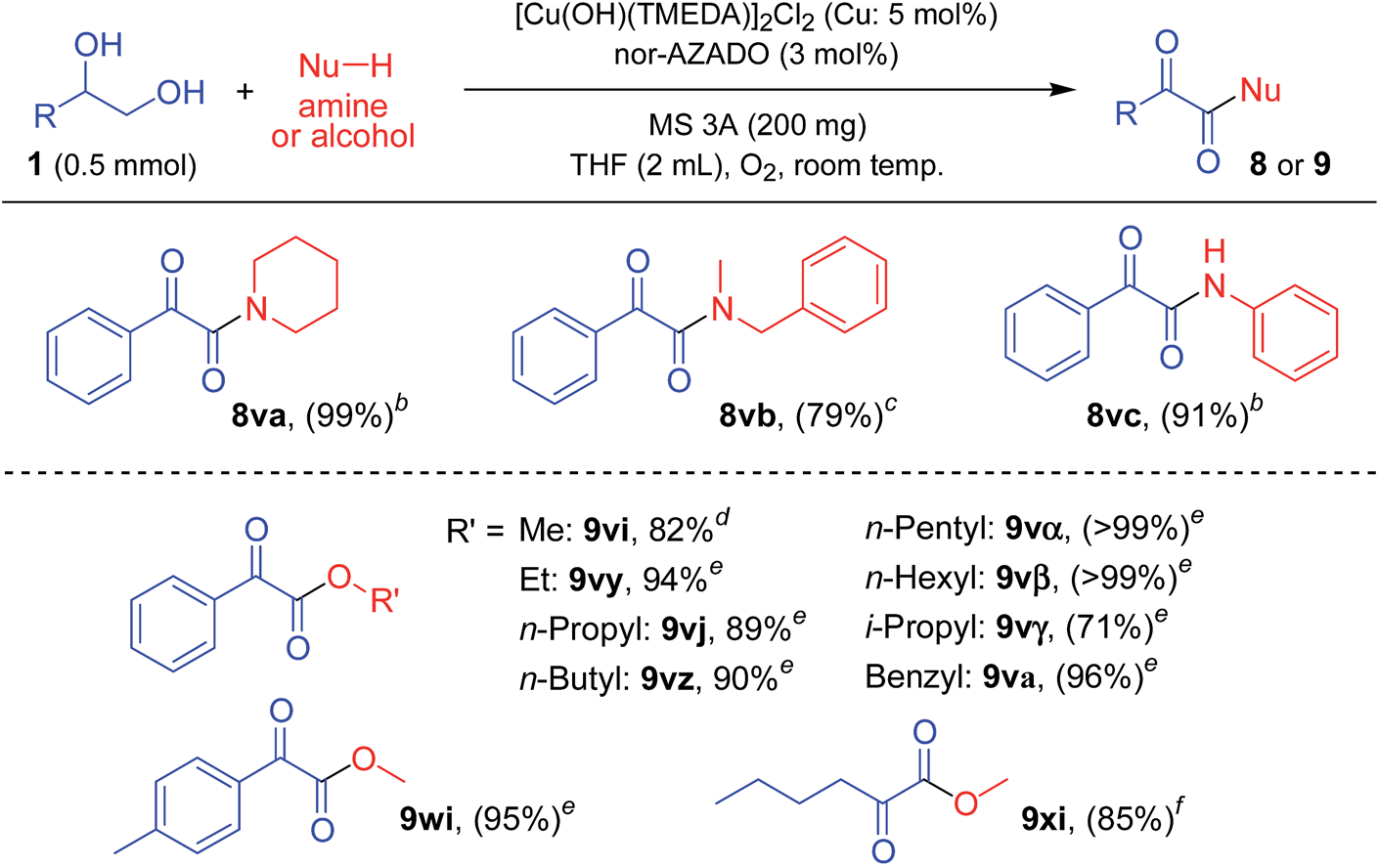

^*a*^Reaction conditions: diol (0.5 mmol), amine (0.6 mmol) or alcohol (2.5 mmol), [Cu(OH)(TMEDA)]_2_Cl_2_ (Cu: 5 mol%), nor-AZADO (3 mol%), MS 3A (200 mg), THF (2 mL), O_2_ (balloon), room temp. (*ca.* 23 °C). Values in parentheses are GC yields.

^*b*^2 h.

^*c*^24 h.

^*d*^5 min.

^*e*^1 h.

^*f*^30 min.

## Conclusion

For the first time, we have successfully developed efficient CuCl/TMEDA/nor-AZADO-catalyzed aerobic oxidative acylation of amides with alcohols. By employing the proposed CuCl/TMEDA/nor-AZADO catalyst system, various kinds of structurally diverse imides, including Evans imides, could be synthesized starting from various combinations of alcohols and amides. In addition, gram-scale production of aniracetam was also successful. The proposed system was also applicable to the synthesis of α-ketocarbonyl compounds from 1,2-diols and nucleophiles, such as amides, amines, and alcohols. One of the most important key points in realizing this efficient acylation system is the utilization of a TMEDA ligand, which, to the best of our knowledge, has never been employed in previously reported Cu/ligand/*N*-oxyl catalyst systems. Based on experimental evidence, such as kinetic and quantum chemical studies, we consider that the plausible roles of TMEDA are the promotion of both hemiamidal oxidation and regeneration of an active Cu^II^–OH species from a Cu^I^ species, and that the promotion of hemiamidal oxidation is crucial to realize efficient acylation. The present catalytic system utilizes O_2_ as the terminal oxidant and produces water as the sole by-product, which highlights its environmentally friendly nature. Moreover, the mild reaction conditions (*e.g.*, room temperature and ambient O_2_ pressure) are matched by excellent functional group tolerance and a very broad substrate scope. We hope that the proposed catalyst system will find broad applications in fine chemical synthesis.

## Experimental section

### Instruments and reagents

GC analyses were performed using a Shimadzu GC-2014 equipped with a flame ionization detector and InertCap-5 capillary column (0.25 mm ID × 30 m length). HPLC analyses were performed on a Shimadzu Prominence system using a UV detector (Shimadzu SPD-20A, 254 nm) equipped with a GL Science Inertsil ODS-3 (4.6 mm ID × 250 mm length) using a methanol/water mixture (9/1 v/v) as an eluent. GC mass spectrometry (GC-MS) spectra were recorded using a Shimadzu GCMS-QP2010 equipped with an InertCap-5 capillary column (0.25 mm ID × 30 m length) at an ionization voltage of 70 eV. Liquid-state NMR spectra were recorded on a JEOL JNM-ECA-500 spectrometer. ^1^H and ^13^C NMR spectra were measured at 500.2 and 125.8 MHz, respectively, using tetramethylsilane as an internal reference (*δ* = 0 ppm). Enantiomeric excess was determined by HPLC analyses performed on a Shimadzu Prominence system using a circular dichroism chiral detector (JASCO CD-2095 Plus, 254 nm) equipped with a Daicel chiralcel OD-H column (4.6 mm ID × 250 mm length) using an *n*-hexane/2-propanol mixture (1/1 v/v) as an eluent. The copper sources, ligands, substrates (alcohols and amides), *N*-oxyls (except for 5-F-AZADO and 4-Cl-AZADO), MS 3A, and solvents were obtained from Kanto Chemical, TCI, Wako, or Aldrich (reagent grade) and purified prior to the use as required. 5-F-AZADO26*a* and 4-Cl-AZADO26*b* were prepared according to the procedures in the literatures. MS 3A was dried under vacuum at 400 °C with a heating mantle before using.

### General procedures for oxidative acylation of amides with alcohols

The reaction was typically carried out according to the following procedures. CuCl (0.025 mmol, 5 mol%), TMEDA (0.025 mmol, 5 mol%), nor-AZADO (0.015 mmol, 3 mol%), THF (2 mL), MS 3A (powder, 200 mg), ^*t*^BuOK (0.5 mmol, if required), and a Teflon-coated magnetic stir bar were placed successively in a Schlenk tube (volume: *ca.* 20 mL), and the reaction mixture was stirred vigorously for 5 min at room temperature (*ca.* 23 °C). The reaction mixture was then purged with O_2_, connected to a balloon filled with O_2_ gas, and stirred vigorously for 2 min at room temperature. Subsequently, a THF solution (0.5 mL) containing alcohol (1.0 M), amide (1.1 M), and an internal standard (diphenyl, 0.2 M) was added to the reaction mixture, which was then vigorously stirred at room temperature. After the reaction was complete, the conversion of substrates and the yields of the products were determined by GC or HPLC analysis. Then, MS 3A was removed by filtration, and the filtrate was concentrated by evaporation. The crude product was subject to column chromatography on silica gel (using a mixture of chloroform/acetone, *n*-hexane/diethyl ether, *n*-hexane/ethyl acetate, or chloroform/methanol as the eluent), giving the pure product. The products were identified by GC-MS and/or NMR (^1^H and ^13^C) analyses.

## Conflicts of interest

There are no conflicts to declare.

## Supplementary Material

Supplementary information
