# Supplementary material for: CuCl/TMEDA/nor-AZADO-catalyzed aerobic oxidative acylation of amides with alcohols to produce imides
Source: Chem Sci. 2018 May 7;9(21):4756–68. doi: 10.1039/c8sc01410h (PMC5982222; doi:10.1039/c8sc01410h)
Supplement: Supplementary file 1 [file SC-009-C8SC01410H-s001.pdf]

Electronic Supplementary Material (ESI) for Chemical Science.
This journal is © The Royal Society of Chemistry 2018

Electronic Supplementary Information (ESI) for

CuCl/TMEDA/nor-AZADO-catalyzed aerobic oxidative acylation of amides with alcohols to produce imides

Kengo Kataoka,[a] Keiju Wachi,[a] Xiongjie Jin,*[b] Kosuke Suzuki,[a] Yusuke Sasano,[c] Yoshiharu Iwabuchi,[c] Jun-ya Hasegawa,[d] Noritaka Mizuno[a] and Kazuya Yamaguchi*[a]

[a] *Department of Applied Chemistry, School of Engineering, The University of Tokyo, 7-3-1 Hongo, Bunkyo-ku, Tokyo 113-8656, Japan. E-mail: kyama@appchem.t.u-tokyo.ac.jp.*
[b] *Department of Chemistry and Biotechnology, School of Engineering, The University of Tokyo, 7-3-1 Hongo, Bunkyo-ku, Tokyo 113-8656, Japan. E-mail: t-jin@mail.ecc.u-tokyo.ac.jp.*
[c] *Department of Organic Chemistry, Graduate School of Pharmaceutical Sciences, Tohoku University, 6-3 Aza-Aoba, Aramaki, Aoba-ku, Sendai 980-8578, Japan.*
[d] *Institute for Catalysis, Hokkaido University, Kita 21 Nishi 10, Kita-ku, Sapporo 001-0021, Japan.*

Spectral Data of Imides

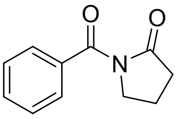

3aa (CAS No. 2399-66-8)

***N*-Benzoyl-2-pyrrolidone (3aa):** ^{1}H NMR (500 MHz, $CDCl_3$, TMS): δ 2.09−2.15 (m, 2H), 2.58 (t, J = 7.5 Hz, 2H), 3.94 (t, J = 7.3 Hz, 2H), 7.39−7.42 (m, 2H), 7.49−7.52 (m, 1H), 7.59−7.61 (m, 2H). ^{13}C{^{1}H} NMR (125 MHz, $CDCl_3$, TMS): δ 17.76, 33.41, 46.65, 127.90, 129.02, 132.03, 134.46, 170.81, 174.70. MS (70 eV, EI): *m*/*z* (%): 189 (20) [M^+], 106 (10), 105 (100), 77 (56), 51 (19).

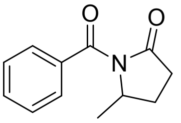

3ab (CAS No. 111607-69-3)

***N*-Benzoyl-5-methyl-2-pyrrolidone (3ab):** ^{1}H NMR (500 MHz, $CDCl_3$, TMS): δ 1.42 (d, J = 6.0 Hz, 3H), 1.72−1.79 (m, 1H), 2.25−2.32 (m, 1H), 2.45−2.51 (m, 1H), 2.61−2.67 (m, 1H), 4.48−4.55 (m, 1H), 7.38−7.42 (m, 2H), 7.49−7.52 (m, 1H), 7.59−7.61 (m, 2H). ^{13}C{^{1}H} NMR (125 MHz, $CDCl_3$, TMS): δ 20.03, 25.94, 31.89, 54.02, 127.96, 128.95, 132.08, 135.08, 170.98, 175.04. MS (70 eV, EI): *m*/*z* (%): 203 (22) [M^+], 202 (21), 105 (100), 98 (27), 77 (34).

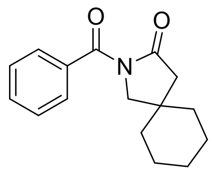

3ac (CAS No. 856069-00-6)

***N*-Benzoyl-4,4-pentamethylene-2-pyrrolidone (3ac):** ^{1}H NMR (500 MHz, $CDCl_3$, TMS): δ 1.48−1.66 (m, 10H), 2.46 (s, 2H), 3.72 (s, 2H), 7.39−7.42 (m, 2H), 7.49−7.52 (m, 1H), 7.58−7.60 (m, 2H). $^{13}C\{^{1}H\}$ NMR (125 MHz, $CDCl_3$, TMS): δ 22.87, 25.73, 35.52, 36.39, 45.89, 57.06, 127.95, 129.03, 132.07, 134.65, 170.90, 174.10. MS (70 eV, EI): *m*/*z* (%): 257 (45) [M^{+}], 152 (40), 106 (13), 105 (100), 77 (31).

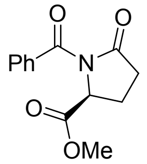

3ad (CAS No. 103322-12-9)

Methyl (*S*)-1-benzoyl-5-oxopyrrolidine-2-carboxylate (3ad): ^{1}H NMR (500 MHz, $CDCl_3$, TMS): δ 2.13−2.19 (m, 1H), 2.41−2.49 (m, 1H), 2.55−2.61 (m, 1H), 2.70−2.77 (m, 1H), 3.81 (s, 3H), 4.89−4.91 (dd, *J* = 9.0 and 3.5 Hz, 1H), 7.40−7.44 (m, 2H), 7.52−7.55 (m, 1H), 7.65−7.67 (m, 2H). $^{13}C\{^{1}H\}$ NMR (125 MHz, $CDCl_3$, TMS): δ 21.98, 31.84, 52.93, 58.81, 128.01, 129.31, 132.48, 133.78, 170.55, 171.71, 173.54. MS (70 eV, EI): *m*/*z* (%): 247 (9) [M^{+}], 188 (23), 105 (100), 77 (39), 51 (11). The enantiomeric excess was >99%*ee*, determined by HPLC analysis (Daicel chiralcel OD-H, *n*-hexane/2-propanol 1:1, 0.5 mL/min). Retention time: 16.6 min.

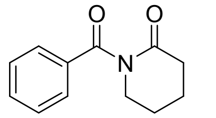

3ae (CAS No. 4252-56-6)

***N*-Benzoyl-2-piperidone (3ae):** ^{1}H NMR (500 MHz, $CDCl_3$, TMS): δ 1.91−2.00 (m, 4H), 2.56 (t, *J* = 6.5 Hz, 2H), 3.80 (t, *J* = 6.0 Hz, 2H), 7.37−7.40 (m, 2H), 7.45−7.49 (m, 1H), 7.54−7.56 (m, 2H). $^{13}C\{^{1}H\}$ NMR (125 MHz, $CDCl_3$, TMS): δ 21.66, 23.01, 34.83, 46.33, 128.05, 128.32, 131.69, 136.29, 173.69, 174.88. MS (70 eV, EI): *m*/*z* (%): 203 (18) [M^{+}], 202 (13), 175 (15), 105 (100), 77 (39).

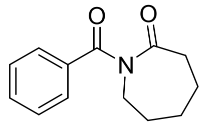

3af (CAS No. 6248-28-8)

***N*-Benzoylazepan-2-one (3af):** ^{1}H NMR (500 MHz, $CDCl_3$, TMS): δ 1.81−1.89 (m, 6H), 2.68−2.71 (m, 2H), 3.96−3.97 (m, 2H), 7.37−7.40 (m, 2H), 7.45−7.48 (m, 1H), 7.53−7.56 (m, 2H). $^{13}C\{^{1}H\}$ NMR (125 MHz, $CDCl_3$, TMS): δ 23.89, 29.37, 29.77, 38.99, 45.31, 127.84, 128.30, 131.48, 136.75, 174.31, 177.75. MS (70 eV, EI): *m*/*z* (%): 217 (18) [M^{+}], 216 (19), 189 (13), 105 (100), 77 (31).

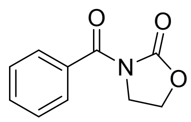

3ag (CAS No. 7007-15-0)

3-Benzoyl-2-oxazolidinone (3ag): MS (70 eV, EI): *m*/*z* (%): 191 (17) [M^+], 105 (100), 77 (32).

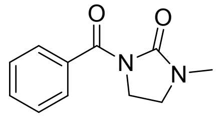

3ah

1-Benzoyl-3-methyl-2-imidazolidinone (3ah): ^{1}H NMR (500 MHz, $CDCl_3$, TMS): δ 2.86 (s, 3H), 3.49−3.52 (m, 2H), 3.99−4.02 (m, 2H), 7.38−7.42 (m, 2H), 7.46−7.50 (m, 1H), 7.56−7.58 (m, 2H). $^{13}C\{^1H\}$ NMR (125 MHz, $CDCl_3$, TMS): δ 30.88, 40.61, 43.30, 127.64, 128.67, 131.28, 134.80, 154.47, 170.52. MS (70 eV, EI): *m*/*z* (%): 204 (28) [M^+], 105 (100), 99 (35), 77 (36).

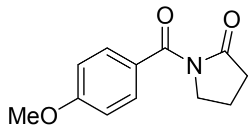

3ba (CAS No. 72432-10-1)

***N*-(4-Methoxybenzoyl)-2-pyrrolidone (3ba):** ^{1}H NMR (500 MHz, $CDCl_3$, TMS): δ 2.09−2.15 (m, 2H), 2.60 (t, J = 8.0 Hz, 2H), 3.85 (s, 3H), 3.93 (t, J = 7.0 Hz, 2H), 6.90 (d, J = 8.5 Hz, 2H), 7.64 (d, J = 8.5 Hz, 2H). $^{13}C\{^1H\}$ NMR (125 MHz, $CDCl_3$, TMS): δ 17.88, 33.56, 46.97, 55.53, 113.23, 126.31, 131.87, 163.04, 170.20, 174.83. MS (70 eV, EI): *m*/*z* (%): 219 (20) [M^+], 135 (100), 92 (12), 77 (15).

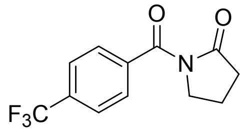

3ca (CAS No. 134407-03-7)

***N*-[4-(trifluoromethyl)benzoyl]-2-pyrrolidone (3ca):** ^{1}H NMR (500 MHz, $CDCl_3$, TMS): δ 2.14−2.20 (m, 2H), 2.62 (t, J = 8.3 Hz, 2H), 3.98 (t, J = 7.0 Hz, 2H), 7.64−7.71 (m, 4H). $^{13}C\{^1H\}$ NMR (125 MHz, $CDCl_3$, TMS): δ 17.75, 33.34, 46.50, 123.87 (q, J = 272.7 Hz), 125.03 (q, J = 3.6 Hz), 129.20, 133.27 (q, J = 32.4 Hz), 138.00, 169.52, 174.80. MS (70 eV, EI): *m*/*z* (%): 257 (25) [M^+], 174 (13), 173 (100), 145 (65), 95 (11), 84 (27).

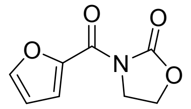

3dg (CAS No. 1394836-09-9)

3-(2-Furoyl)-2-oxazolidinone (3dg): MS (70 eV, EI): *m*/*z* (%): 181 (20) [M^+], 95 (100).

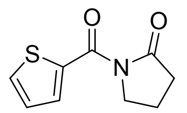

3ea (CAS No. 1394836-00-0)

1-(Thiophene-2-carbonyl)-2-pyrrolidone (3ea): MS (70 eV, EI): *m*/*z* (%): 195 (24) [M^+], 111 (100), 84 (11).

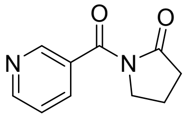

3fa (CAS No. 34236-73-2)

***N*-(3-Pyridinylcarbonyl)-2-pyrrolidone (3fa):** MS (70 eV, EI): *m*/*z* (%): 190 (38) [M^+], 162 (20), 107 (16), 106 (100), 84 (14), 79 (24), 78 (75), 51 (36), 50 (11).

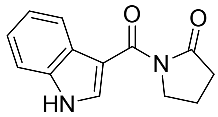

3ga

3-[(2-Pyrrolidonyl)carbonyl]indole (3ga): ^{1}H NMR (500 MHz, $(CD_3)_2SO$, TMS): δ 1.98−2.04 (m, 2H), 2.55 (t, J = 7.8 Hz, 2H), 3.82 (t, J = 7.0 Hz, 2H), 7.16−7.22 (m, 2H), 7.48−7.49 (m, 1H), 8.01−8.02 (m, 1H), 8.11−8.12 (m, 1H), 11.95 (brs, 1H). ^{13}C{^{1}H} NMR (125 MHz, $(CD_3)_2SO$, TMS): δ 17.47, 33.20, 46.72, 108.83, 112.21, 120.71, 121.43, 122.38, 126.73, 135.22, 135.80, 164.85, 174.46. MS (70 eV, EI): *m*/*z* (%): 228 (32) [M^+], 145 (11), 144 (100), 116 (20), 89 (16).

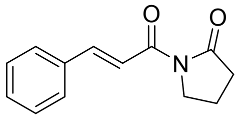

3ha (CAS No. 141236-49-9)

***N*-Cinnamoyl-2-pyrrolidone (3ha):** MS (70 eV, EI): *m*/*z* (%): 215 (35) [M^+], 132 (17), 131 (100), 104 (10), 103 (34), 77 (15).

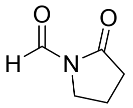

3ia (CAS No. 40321-44-6)

***N*-Formyl-2-pyrrolidone (3ia):** MS (70 eV, EI): *m*/*z* (%): 113 (11) [M^+], 85 (100), 84 (18), 57 (27), 56 (22).

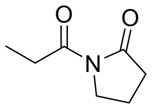

3ja (CAS No. 77015-41-9)

***N*-Propionyl-2-pyrrolidone (3ja):** ^{1}H NMR (500 MHz, $CDCl_3$, TMS): δ 1.15 (t, J = 7.5 Hz, 3H), 2.01−2.07 (m, 2H), 2.60 (t, J = 8.0 Hz, 2H), 2.91 (q, J = 7.5 Hz, 2H), 3.82 (t, J = 7.3 Hz, 2H). ^{13}C{^{1}H} NMR (125 MHz, $CDCl_3$, TMS): δ 8.48, 17.46, 30.61, 33.92, 45.68, 175.39, 175.61. MS (70 eV, EI): *m*/*z* (%): 141 (48) [M^+], 113 (40), 112 (15), 98 (17), 86 (90), 85 (13), 84 (15), 69 (17), 57 (100), 56 (22).

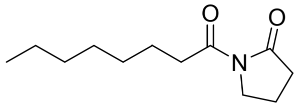

3ka (CAS No. 60437-60-7)

***N*-Octanoyl-2-pyrrolidone (3ka):** ^{1}H NMR (500 MHz, $CDCl_3$, TMS): δ 0.88 (t, J = 6.8 Hz, 3H), 1.28−1.38 (m, 8H), 1.60−1.66 (m, 2H), 2.00−2.06 (m, 2H), 2.60 (t, J = 8.0 Hz, 2H), 2.89 (t, J = 7.5 Hz, 2H), 3.81 (t, J = 7.0 Hz, 2H). $^{13}C\{^{1}H\}$ NMR (125 MHz, $CDCl_3$, TMS): δ 14.26, 17.38, 22.79, 24.39, 29.24, 29.34, 31.87, 33.93, 37.00, 45.64, 174.71, 175.50. MS (70 eV, EI): *m*/*z* (%): 211 (1) [M^+], 140 (74), 127 (81), 99 (47), 98 (22), 86 (100), 85 (15), 84 (13), 69 (14), 57 (44), 56 (18), 55 (43).

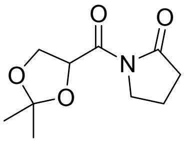

3la

***N*-[(2,2-Dimethyl-1,3-dioxolan-4-yl)carbonyl]-2-pyrrolidone (3la):** ^{1}H NMR (500 MHz, $CDCl_3$, TMS): δ 1.42 (s, 3H), 1.58 (s, 3H), 2.05−2.18 (m, 2H), 2.56−2.67 (m, 2H), 3.80−3.88 (m, 2H), 4.02−4.05 (dd, J = 9.5 and 4.0 Hz, 1H), 4.40−4.43 (dd, J = 9.3 and 7.3 Hz, 1H), 5.38−5.40 (dd, J = 7.3 and 3.8 Hz, 1H). $^{13}C\{^{1}H\}$ NMR (125 MHz, CDCl3, TMS): δ 17.94, 25.63, 26.25, 33.39, 45.51, 68.03, 76.55, 111.68, 172.13, 176.10. MS (70 eV, EI): *m*/*z* (%): 198 (40), 155 (33), 138 (64), 101 (100), 100 (10), 86 (57), 85 (19), 83 (11), 73 (30), 69 (11), 56 (18), 55 (14).

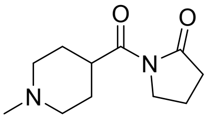

3ma

1-Methyl-4-[(2-pyrrolidonyl)carbonyl]piperidine (3ma): ^{1}H NMR (500 MHz, $CDCl_3$, TMS): δ 1.73−1.85 (m, 4H), 2.01−2.07 (m, 4H), 2.27 (s, 3H), 2.61 (t, J = 8.0 Hz, 2H), 2.87−2.90 (m, 2H), 3.38−3.44 (m, 1H), 3.79−3.82 (m, 2H). $^{13}C\{^{1}H\}$ NMR (125 MHz, $CDCl_3$, TMS): δ 17.31, 28.36, 34.11, 41.28, 45.98, 46.50, 55.24, 175.21, 176.72. MS (70 eV, EI): *m*/*z* (%): 210 (33) [M^+], 140 (46), 125 (18), 98 (56), 97 (65), 96 (73), 86 (18), 82 (22), 71 (70), 70 (100), 69 (11), 68 (11), 58 (14), 57 (18), 56 (18), 55 (45).

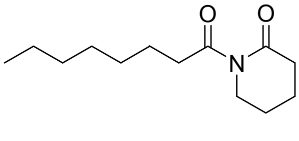

3ke (CAS No. 60437-61-8)

***N*-Octanoyl-2-piperidone (3ke):** ^{1}H NMR (500 MHz, $CDCl_3$, TMS): δ 0.88 (t, J = 6.8 Hz, 3H), 1.24−1.35 (m, 8H), 1.60−1.66 (m, 2H), 1.79−1.87 (m, 4H), 2.54−2.56 (m, 2H), 2.89 (t, J = 7.5 Hz, 2H), 3.72 (t, J = 6.0 Hz, 2H). $^{13}C\{^{1}H\}$ NMR (125 MHz, $CDCl_3$, TMS): δ 14.27, 20.47, 22.65, 22.80, 25.13, 29.29, 29.39, 31.90, 35.10, 39.79, 44.05, 173.52, 177.38. MS (70 eV, EI): *m*/*z* (%): 225 (2) [M^+], 154 (40), 141 (25), 113 (15), 100 (100), 99 (38), 98 (11), 57 (23), 55 (17).

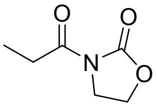

3jg (CAS No. 60420-27-1)

3-Propionyl-2-oxazolidinone (3jg): MS (70 eV, EI): *m*/*z* (%): 143 (9) [M^+], 115 (24), 88 (51), 57 (100), 56 (15).

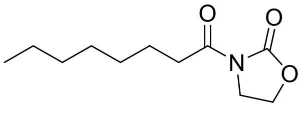

3kg (CAS No. 60420-32-8)

3-Octanoyl-2-oxazolidinone (3kg): ^{1}H NMR (500 MHz, $CDCl_3$, TMS): δ 0.88 (t, J = 7.0 Hz, 3H), 1.26−1.38 (m, 8H), 1.63−1.69 (m, 2H), 2.91 (t, J = 7.8 Hz, 2H), 4.02 (t, J = 7.8 Hz, 2H), 4.41 (t, J = 7.0 Hz, 2H). $^{13}C\{^1H\}$ NMR (125 MHz, $CDCl_3$, TMS): δ 14.27, 22.79, 24.43, 29.21, 29.25, 31.85, 35.29, 42.70, 62.18, 153.74, 173.81. MS (70 eV, EI): *m*/*z* (%): 213 (0.4) [M^+], 142 (64), 129 (100), 127 (24), 101 (24), 88 (46), 57 (45), 55 (20).

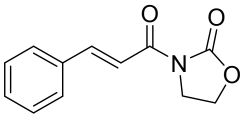

3hg (CAS No. 109299-93-6)

3-Cinnamoyl-2-oxazolidinone (3hg): ^{1}H NMR (500 MHz, $CDCl_3$, TMS): δ 4.13 (t, J = 8.0 Hz, 2H), 4.46 (t, J = 8.0 Hz, 2H), 7.39−7.41 (m, 3H), 7.61−7.64 (m, 2H), 7.85−7.93 (m, 2H). $^{13}C\{^1H\}$ NMR (125 MHz, $CDCl_3$, TMS): δ 43.00, 62.27, 116.75, 128.81, 129.07, 130.87, 134.67, 146.47, 153.79, 165.56. MS (70 eV, EI): *m*/*z* (%): 217 (25) [M^+], 132 (11), 131 (100), 103 (45), 102 (14), 77 (26), 51 (12).

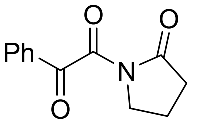

3va (CAS No. 2399-66-8)

1-(2-Oxo-1-pyrrolidinyl)-2-phenyl-1,2-ethanedione (3va): MS (70 eV, EI): *m*/*z* (%): 217 (1) [M^+], 189 (15), 105 (100), 77 (44), 51 (16).

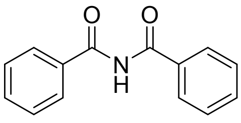

3ai (CAS No. 614-28-8)

***N*-Benzoylbenzamide (3ai):** ^{1}H NMR (500 MHz, $CDCl_3$, TMS): δ 7.51 (t, J = 7.5 Hz, 4H), 7.61 (t, J = 7.5 Hz, 2H), 7.86−7.88 (m, 4H), 8.98 (brs, 1H). $^{13}C\{^1H\}$ NMR (125 MHz, $CDCl_3$, TMS): δ 128.13, 129.10, 133.33, 133.54, 166.55. MS (70 eV, EI): *m*/*z* (%): 225 (16) [M^+], 122 (11), 105 (100), 103 (26), 77 (69), 76 (13), 51 (31), 50 (14).

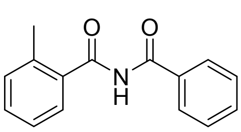

3ni (CAS No. 80791-90-8)

***N*-Benzoyl-2-methylbenzamide (3ni):** ^{1}H NMR (500 MHz, $CDCl_3$, TMS): δ 2.47 (s, 3H), 7.25−7.27 (m, 2H),

7.37−7.40 (dt, J = 1.3 and 7.6 Hz, 1H), 7.44−7.50 (m, 3H), 7.58−7.61 (m, 1H), 7.87−7.89 (m, 2H), 9.11 (brs, 1H). $^{13}C\{^1H\}$ NMR (125 MHz, $CDCl_3$, TMS): δ 20.02, 125.98, 127.15, 128.13, 129.07, 131.17, 131.41, 132.97, 133.41, 134.90, 136.85, 165.78, 170.10.

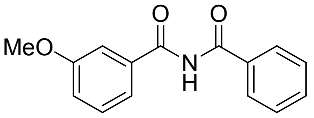

3oi and 3ak (CAS No. 1776069-60-3)

***N*-Benzoyl-3-methoxybenzamide (3oi and 3ak):** ^1H NMR (500 MHz, $CDCl_3$, TMS): δ 3.81 (s, 3H), 7.08−7.10 (dd, J = 8.0 and 2.0 Hz, 1H), 7.35 (t, J = 8.0 Hz, 1H), 7.39−7.40 (m, 2H), 7.45 (t, J = 7.5 Hz, 2H), 7.56 (t, J = 7.5 Hz, 1H), 7.84 (d, J = 7.5 Hz, 2H), 9.30 (brs, 1H). $^{13}C\{^1H\}$ NMR (125 MHz, $CDCl_3$, TMS): δ 55.56, 113.24, 119.28, 119.80, 128.09, 128.80, 129.86, 133.05, 133.43, 134.77, 159.93, 166.25, 166.74.

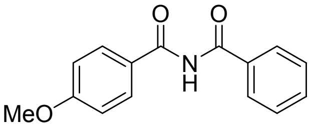

3bi and 3aj (CAS No. 77290-63-2)

***N*-Benzoyl-4-methoxybenzamide (3bi and 3aj):** ^1H NMR (500 MHz, $CDCl_3$, TMS): δ 3.84 (s, 3H), 6.93 (d, J = 8.5 Hz, 2H), 7.45 (t, J = 7.5 Hz, 2H), 7.56 (t, J = 7.3 Hz, 1H), 7.83−7.85 (m, 4H), 9.20 (brs, 1H). $^{13}C\{^1H\}$ NMR (125 MHz, $CDCl_3$, TMS): δ 55.55, 114.01, 125.39, 128.06, 128.74, 130.42, 132.89, 133.59, 163.50, 166.02, 166.99.

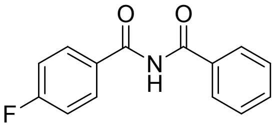

3pi (CAS No. 866353-52-8)

***N*-Benzoyl-4-fluorobenzamide (3pi):** ^1H NMR (500 MHz, $CDCl_3$, TMS): δ 7.09−7.12 (m, 2H), 7.42−7.45 (m, 2H), 7.53−7.56 (m, 1H), 7.83−7.90 (m, 4H), 9.47 (brs, 1H). $^{13}C\{^1H\}$ NMR (125 MHz, $CDCl_3$, TMS): δ 115.71 (d, J = 21.6 Hz), 128.07, 128.61, 129.39, 130.87 (d, J = 8.4 Hz), 132.96, 133.08, 165.43 (d, J = 254.3 Hz), 166.00, 166.86.

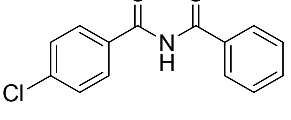

3qi and 3am (CAS No. 58010-69-8)

***N*-Benzoyl-4-chlorobenzamide (3qi and 3am):** ^1H NMR (500 MHz, $CDCl_3$, TMS): δ 7.34−7.45 (m, 4H), 7.54 (t, J = 7.5 Hz, 1H), 7.78 (d, J = 8.0 Hz, 2H), 7.83 (d, J = 8.0 Hz, 2H), 9.54 (brs, 1H). $^{13}C\{^1H\}$ NMR (125 MHz, $CDCl_3$, TMS): δ 128.21, 128.79, 128.99, 129.79, 131.77, 133.14, 133.19, 139.40, 166.39, 166.94.

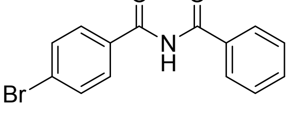

3ri (CAS No. 1590379-99-9)

***N*-Benzoyl-4-bromobenzamide (3ri):** ^{1}H NMR (500 MHz, $CDCl_3$, TMS): δ 7.44 (t, J = 7.5 Hz, 2H), 7.55−7.57 (m, 3H), 7.70 (d, J = 8.0 Hz, 2H), 7.84 (d, J = 8.0 Hz, 2H), 9.43 (brs, 1H). ^{13}C{^{1}H} NMR (125 MHz, $CDCl_3$, TMS): δ 128.05, 128.20, 128.85, 129.87, 132.02, 132.25, 133.12, 133.25, 166.55, 166.84.

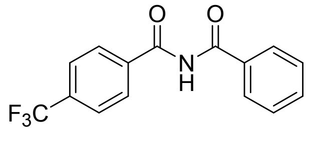

3ci

***N*-Benzoyl-4-(trifluoromethyl)benzamide (3ci):** ^{1}H NMR (500 MHz, $CDCl_3$, TMS): δ 7.48−7.51 (m, 2H), 7.59−7.63 (m, 1H), 7.72 (d, J = 8.0 Hz, 2H), 7.86−7.89 (m, 2H), 7.94 (d, J = 8.5 Hz, 2H), 9.32 (brs, 1H). ^{13}C{^{1}H} NMR (125 MHz, $CDCl_3$, TMS): δ 123.58 (q, J = 272.7 Hz), 125.74 (q, J = 3.6 Hz), 128.29, 128.78, 128.93, 132.91, 133.46, 134.31 (q, J = 32.8 Hz), 136.85, 166.78, 166.84.

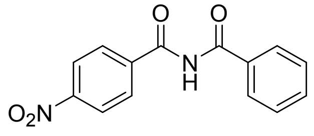

3si (CAS No. 39057-66-4)

***N*-Benzoyl-4-nitrobenzamide (3si):** ^{1}H NMR (500 MHz, $(CD_3)_2SO$, TMS): δ 7.54−7.57 (t, J = 7.3 Hz, 2H), 7.65−7.68 (t, J = 7.3 Hz, 1H), 7.96 (d, J = 7.0 Hz, 2H), 8.11 (d, J = 8.0 Hz, 2H), 8.34 (d, J = 8.0 Hz, 2H), 11.65 (brs, 1H). ^{13}C{^{1}H} NMR (125 MHz, $(CD_3)_2SO$, TMS): δ 123.42, 128.45, 128.76, 129.90, 132.94, 133.27, 139.86, 149.42, 167.07, 167.41.

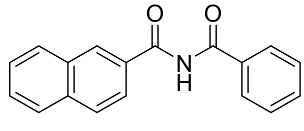

3ti (CAS No. 130956-01-3)

***N*-Benzoyl-2-naphthamide (3ti):** ^{1}H NMR (500 MHz, $CDCl_3$, TMS): δ 7.44 (t, J = 7.5 Hz, 2H), 7.48−7.51 (m, 1H), 7.53−7.57 (m, 2H), 7.81−7.83 (m, 3H), 7.85−7.88 (m, 3H), 8.33 (s, 1H), 9.46 (brs, 1H). ^{13}C{^{1}H} NMR (125 MHz, $CDCl_3$, TMS): δ 124.00, 127.00, 127.79, 128.14, 128.48, 128.65, 128.75, 129.21, 129.24, 130.50, 132.30, 132.99, 133.36, 135.32, 166.78, 166.80.

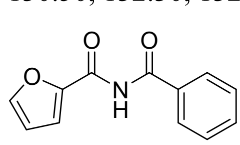

3di (CAS No. 89549-39-3)

***N*-Benzoyl-2-furancarboxamide (3di):** ^{1}H NMR (500 MHz, $CDCl_3$, TMS): δ 6.63−6.64 (dd, J = 3.5 and 2.0 Hz, 1H), 7.40−7.41 (dd, J = 3.5 and 1.0 Hz, 1H), 7.57−7.58 (dd, J = 1.5 and 0.5 Hz, 1H), 7.51−7.55 (m, 2H), 7.61−7.64 (tt, J = 7.5 and 1.4 Hz, 1H), 7.89−7.91 (m, 2H), 9.31 (brs, 1H). ^{13}C{^{1}H} NMR (125 MHz, $CDCl_3$, TMS): δ 113.47, 118.22, 127.92, 129.07, 133.29, 133.46, 145.49, 146.70, 155.12, 165.32.

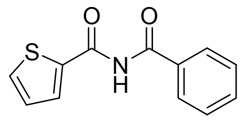

3ei (CAS No. 1590380-00-9)

***N*-Benzoyl-2-thiophenecarboxamide (3ei):** ^{1}H NMR (500 MHz, $CDCl_3$, TMS): δ 7.12−7.14 (m, 1H), 7.43−7.47 (m, 2H), 7.54−7.57 (m, 1H), 7.63−7.65 (m, 1H), 7.81−7.86 (m, 3H), 9.24 (brs, 1H). ^{13}C{^{1}H} NMR (125 MHz, $CDCl_3$, TMS): δ 128.18, 128.32, 128.87, 131.47, 133.13, 133.54, 133.65, 137.78, 160.24, 166.79.

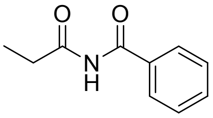

3ji (CAS No. 28358-79-4)

***N*-Propionylbenzamide (3ji):** MS (70 eV, EI): *m*/*z* (%): 177 (29) [M^+], 149 (15), 122 (27), 105 (100), 77 (33), 57 (12).

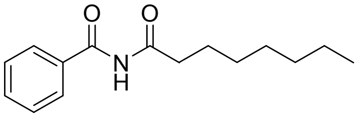

3ki and 3as (CAS No. 695203-41-9)

***N*-Octanoylbenzamide (3ki and 3as):** MS (70 eV, EI): *m*/*z* (%): 247 (3) [M^+], 176 (27), 163 (32), 162 (14), 122 (36), 105 (100), 77 (44), 57 (14), 55 (12), 51 (10).

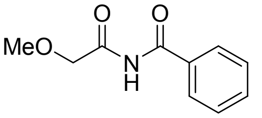

3ui (CAS No. 959095-81-9)

***N*-(2-Methoxyacetyl)-benzamide (3ui):** ^{1}H NMR (500 MHz, $CDCl_3$, TMS): δ 3.53 (s, 3H), 4.40 (s, 2H), 7.49−7.53 (m, 2H), 7.60−7.63 (m, 1H), 7.87−7.89 (m, 2H), 9.42 (brs, 1H). ^{13}C{^{1}H} NMR (125 MHz, $CDCl_3$, TMS): δ 59.58, 73.13, 127.94, 129.16, 132.57, 133.52, 165.29, 171.14. MS (70 eV, EI): *m*/*z* (%): 193 (5) [M^+], 178 (11), 163 (16), 105 (100), 77 (54), 51 (19).

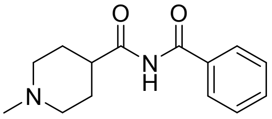

3mi

***N*-Benzoyl-1-methyl-4-piperidinecarboxamide (3mi):** ^{1}H NMR (500 MHz, $CDCl_3$, TMS): δ 1.79−1.87 (m, 2H), 1.96−1.99 (m, 2H), 2.04−2.10 (m, 2H), 2.29 (s, 3H), 2.90−2.93 (m, 2H), 3.37−3.43 (tt, J = 11.3 and 3.7 Hz, 1H), 7.48−7.51 (m, 2H), 7.58−7.62 (m, 1H), 7.91−7.93 (m, 2H), 9.32 (brs, 1H). ^{13}C{^{1}H} NMR (125 MHz, $CDCl_3$, TMS): δ 28.36, 42.41, 46.55, 55.26, 128.00, 129.02, 132.95, 133.32, 165.83, 178.92. MS (70 eV, EI): *m*/*z* (%): 246 (18) [M^+], 176 (19), 105 (43), 98 (20), 97 (55), 96 (100), 82 (13), 77 (31), 71 (27), 70 (60), 57 (10), 55 (25), 51 (11).

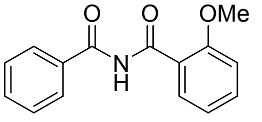

3al (CAS No. 92552-95-9)

***N*-Benzoyl-2-methoxybenzamide (3al):** ^{1}H NMR (500 MHz, $CDCl_3$, TMS): δ 4.09 (s, 3H), 7.06 (d, J = 8.0 Hz, 1H), 7.10−7.13 (m, 1H), 7.50−7.56 (m, 3H), 7.58−7.61 (m, 1H), 7.89−7.90 (m, 2H), 8.25−8.27 (dd, J = 8.0 and 1.5 Hz, 1H), 11.23 (brs, 1H). ^{13}C{^{1}H} NMR (125 MHz, $CDCl_3$, TMS): δ 56.66, 111.80, 120.72, 122.03, 127.49, 128.97, 132.88, 133.24, 134.17, 134.69, 157.37, 162.93, 165.19.

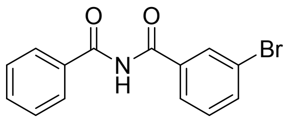

3an (CAS No. 109312-13-2)

***N*-Benzoyl-3-bromobenzamide (3an):** ^{1}H NMR (500 MHz, $CDCl_3$, TMS): δ 7.32 (t, J = 8.0 Hz, 1H), 7.46 (t, J = 7.8 Hz, 2H), 7.57 (t, J = 7.3 Hz, 1H), 7.65−7.67 (m, 1H), 7.76 (d, J = 8.0 Hz, 1H), 7.84−7.86 (m, 2H), 7.96−7.97 (m, 1H), 9.40 (brs, 1H). ^{13}C{^{1}H} NMR (125 MHz, $CDCl_3$, TMS): δ 122.96, 126.81, 128.28, 128.95, 130.38, 131.36, 133.14, 133.37, 135.47, 135.99, 165.92, 166.79.

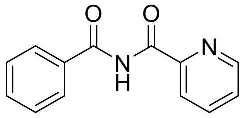

3ao (CAS No. 956028-68-5)

***N*-Benzoyl-2-pyridinecarboxamide (3ao):** ^{1}H NMR (500 MHz, $CDCl_3$, TMS): δ 7.53−7.65 (m, 4H), 7.94−8.02 (m, 3H), 8.33−8.35 (m, 1H), 8.66−8.67 (m, 1H), 11.55 (brs, 1H). ^{13}C{^{1}H} NMR (125 MHz, $CDCl_3$, TMS): δ 123.44, 127.91, 127.96, 129.10, 133.25, 133.57, 138.22, 148.46, 148.69, 162.13, 164.96.

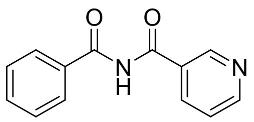

3ap (CAS No. 756488-91-2)

***N*-Benzoyl-3-pyridinecarboxamide (3ap):** ^{1}H NMR (500 MHz, $CDCl_3$, TMS): δ 7.35−7.38 (m, 1H), 7.42−7.46 (m, 2H), 7.54−7.57 (m, 1H), 7.86−7.88 (m, 2H), 8.12−8.14 (m, 1H), 8.65−8.67 (m, 1H), 8.99 (m, 1H), 10.04 (brs, 1H). ^{13}C{^{1}H} NMR (125 MHz, $CDCl_3$, TMS): δ 123.58, 128.37, 128.90, 129.81, 132.97, 133.44, 136.50, 149.20, 153.08, 166.80, 167.10.

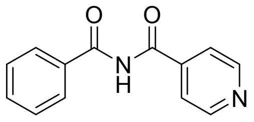

3aq

***N*-Benzoyl-4-pyridinecarboxamide (3aq):** ^{1}H NMR (500 MHz, $(CD_3)_2SO$, TMS): δ 7.53−7.56 (m, 2H), 7.65−7.68 (m, 1H), 7.78−7.79 (m, 2H), 7.95−7.97 (m, 2H), 8.78−8.79 (m, 2H), 11.61 (brs, 1H). ^{13}C{^{1}H} NMR (125 MHz, $(CD_3)_2SO$, TMS): δ 121.97, 128.46, 128.78, 132.96, 133.27, 141.34, 150.16, 167.12, 167.36.

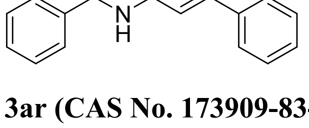

3ar (CAS No. 173909-83-6)

***N*-Cinnamoylbenzamide (3ar):** ^{1}H NMR (500 MHz, $CDCl_3$, TMS): δ 7.40−7.42 (m, 3H), 7.52 (t, J = 7.8 Hz, 2H), 7.56−7.65 (m, 3H), 7.84−7.97 (m, 4H), 9.28 (brs, 1H). $^{13}C\{^1H\}$ NMR (125 MHz, $CDCl_3$, TMS): δ 119.65, 128.11, 128.82, 129.07, 129.09, 130.87, 133.14, 133.40, 134.76, 146.82, 166.31, 168.23.

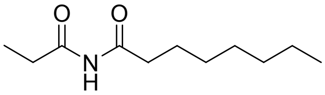

3js (CAS No. 109777-03-9)

***N*-Propionyloctaneamide (3js):** ^{1}H NMR (500 MHz, $CDCl_3$, TMS): δ 0.88 (t, J = 7.0 Hz, 3H), 1.16 (t, J = 7.5 Hz, 3H), 1.28−1.36 (m, 8H), 1.62−1.68 (m, 2H), 2.58−2.68 (m, 4H), 9.13 (brs, 1H). $^{13}C\{^1H\}$ NMR (125 MHz, $CDCl_3$, TMS): δ 8.53, 14.23, 22.77, 24.62, 29.17, 29.25, 30.90, 31.84, 37.58, 174.94, 175.83. MS (70 eV, EI): *m*/*z* (%): 199 (3) [M^+], 156 (11), 144 (15), 142 (11), 128 (61), 127 (22), 126 (11), 115 (100), 98 (10), 87 (24), 86 (10), 74 (62), 73 (95), 72 (47), 60 (12), 59 (64), 57 (100), 55 (21).

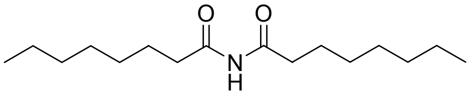

3ks (CAS No. 13916-42-2)

***N*-Octanoyloctaneamide (3ks):** ^{1}H NMR (500 MHz, $CDCl_3$, TMS): δ 0.88 (t, J = 7.0 Hz, 6H), 1.28−1.36 (m, 16H), 1.61−1.67 (m, 4H), 2.60 (t, J = 2.3 Hz, 4H), 8.89 (brs, 1H). $^{13}C\{^1H\}$ NMR (125 MHz, $CDCl_3$, TMS): δ 14.26, 22.80, 24.59, 29.21, 29.26, 31.87, 37.63, 174.90. MS (70 eV, EI): *m*/*z* (%): 269 (10) [M^+], 226 (22), 212 (12), 198 (60), 185 (25), 144 (100), 143 (17), 142 (19), 128 (17), 127 (63), 126 (61), 114 (49), 101 (20), 100 (21), 98 (21), 86 (26), 84 (14), 73 (11), 72 (99), 60 (31), 59 (53), 57 (78), 55 (28).

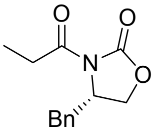

3jt (CAS No. 101711-78-8)

(*S*)-4-Benzyl-3-propionyl-2-oxazolidinone (3jt): ^{1}H NMR (500 MHz, $CDCl_3$, TMS): δ 1.21 (t, J = 7.3 Hz, 3H), 2.75−2.80 (dd, J = 13.3 and 9.8 Hz, 1H), 2.89−3.03 (m, 2H), 3.29−3.32 (dd, J = 13.5 and 3.5 Hz, 1H), 4.16−4.22 (m, 2H), 4.65−4.70 (m, 1H), 7.21−7.22 (m, 2H), 7.26−7.29 (m, 1H), 7.32−7.35 (m, 2H). $^{13}C\{^1H\}$ NMR (125 MHz, $CDCl_3$, TMS): δ 8.48, 29.40, 38.10, 55.36, 66.40, 127.53, 129.15, 129.61, 135.52, 153.71, 174.27. MS (70 eV, EI): *m*/*z* (%): 233 (14) [M^+], 142 (24), 91 (19), 57 (100). The enantiomeric excess was >99%*ee*, determined by HPLC analysis (Daicel chiralcel OD-H, *n*-hexane/2-propanol 1:1, 0.5 mL/min). Retention time: 12.6 min.

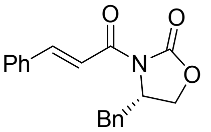

3ht (CAS No. 128891-66-7)

(*S*)-4-Benzyl-3-cinnamoyl-2-oxazolidinone (3ht): ^{1}H NMR (500 MHz, $CDCl_3$, TMS): δ 2.83−2.88 (dd, J = 13.3 and 9.3 Hz, 1H), 3.36−3.39 (dd, J = 13.3 and 3.3 Hz, 1H), 4.20−4.27 (m, 2H), 4.78−4.83 (m, 1H), 7.24−7.42 (m, 8H), 7.62−7.66 (m, 2H), 7.89−7.96 (m, 2H). $^{13}C\{^1H\}$ NMR (125 MHz, $CDCl_3$, TMS): δ 38.11, 55.62, 66.37, 117.16, 127.54, 128.88, 129.10, 129.17, 129.67, 130.94, 134.72, 135.53, 146.68, 153.74, 165.40.

MS (70 eV, EI): m/z (%): 307 (17) [M^+], 132 (10), 131 (100), 103 (31), 77 (18). The enantiomeric excess was >99%*ee*, determined by HPLC analysis (Daicel chiralcel OD-H, *n*-hexane/2-propanol 1:1, 0.5 mL/min). Retention time: 18.9 min.

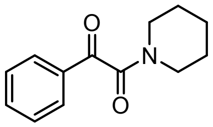

8va (CAS No. 14377-63-0)

1-(Phenylglyoxylyl)piperidine (8va): MS (70 eV, EI): m/z (%): 217 (6) [M^+], 112 (100), 105 (54), 84 (18), 77 (38), 69 (57), 51 (14).

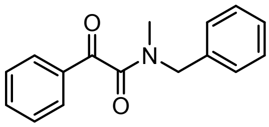

8vb (CAS No. 95725-09-0)

***N*-Benzyl-*N*-methyl-2-oxo-2-phenylacetamide (8vb):** MS (70 eV, EI): m/z (%): 253 (3) [M^+], 148 (10), 120 (54), 105 (89), 91 (100), 77 (45), 65 (12), 51 (14).

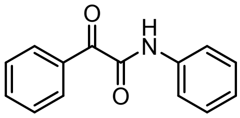

8vc (CAS No. 4732-66-5)

2-Oxo-*N*,2-diphenylacetamide (8vc): MS (70 eV, EI): m/z (%): 225 (22) [M^+], 119 (10), 105 (100), 77(52), 51 (15).

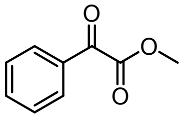

9vi (CAS No. 15206-55-0)

Methyl phenylglyoxylate (9vi):^{1}H NMR (500 MHz, $CDCl_3$, TMS): δ 3.99 (s, 3H), 7.51–7.54 (m, 2H), 7.66–7.69 (m, 1H), 8.02–8.03 (m, 2H). ^{13}C NMR (125 MHz, $CDCl_3$, TMS): δ 52.81, 128.92, 130.10, 132.41, 135.02, 164.04, 186.06. MS (70 eV, EI): m/z (%): 164 (1) [M^+], 105 (100), 77 (62), 51 (25).

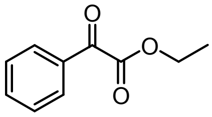

9vy (CAS No. 1603-79-8)

Ethyl phenylglyoxylate (9vy): ^{1}H NMR (500MHz, $CDCl_3$, TMS): δ 1.43 (t, J = 7.3 Hz, 3H), 4.46 (q, J = 7.2 Hz, 2H), 7.51–7.54 (m, 2H), 7.65–7.68 (m, 1H), 8.01–8.02 (m, 2H). ^{13}C NMR (125 MHz, $CDCl_3$, TMS): δ 14.14, 62.37, 128.90, 130.56, 132.47, 134.92, 163.84, 186.44. MS (70 eV, EI): m/z (%): 178 (0.5) [M^+], 105 (100), 77 (49), 51 (20).

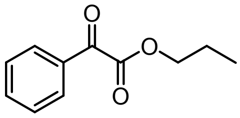

9vj (CAS No. 31197-63-4)

***n*-Propyl phenylglyozylate (9vj):** ^{1}H NMR (500 MHz, $CDCl_3$, TMS): δ 1.02 (t, *J* = 7.3 Hz, 3H), 1.82 (sext, *J* = 7.1 Hz, 2H), 4.36 (t, *J* = 6.8 Hz, 2H), 7.51–7.54 (m, 2H), 7.65–7.68 (m, 1H), 8.00–8.02 (m, 2H). ^{13}C NMR (125 MHz, $CDCl_3$, TMS): δ 10.32, 21.89, 67.79, 128.91, 130.02, 132.49, 134.91, 164.02, 186.51. MS (70 eV, EI): *m*/*z* (%): 192 (0.4) [M^+], 105 (100), 77 (42), 51 (13).

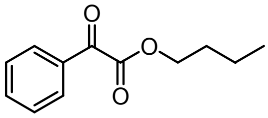

9vz (CAS No. 5524-55-0)

***n*-Butyl phenylglyoxylate (9vz):** ^{1}H NMR (500 MHz, $CDCl_3$, TMS): δ 0.97 (t, *J* = 7.3, 3H), 1.46 (sext, *J* = 7.5, 2H), 1.77 (quin, *J* = 7.1, 2H), 4.40 (t, *J* = 6.8, 2H), 7.51–7.54 (m, 2H), 7.67–7.68 (m, 1H), 8.00–8.02(m, 2H). ^{13}C NMR (125 MHz, $CDCl_3$, TMS): δ 13.65, 19.04, 30.47, 66.12, 128.90, 130.02, 132.49, 134.90, 164.02, 186.51. MS (70 eV, EI): *m*/*z* (%): 206 (0.4) [M^+], 105 (100), 77 (35), 51 (12).

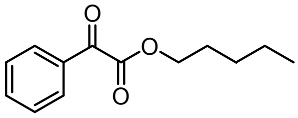

9vα (CAS No. 31197-64-5)

Pentyl phenylglyoxylate (9vα): MS (70 eV, EI): *m*/*z* (%): 220 (0.3) [M^+], 105 (100), 77 (33), 51 (10).

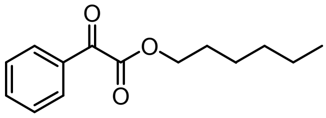

9vβ (CAS No. 31197-65-6)

Hexyl phenylglyoxylate (9vβ): MS (70 eV, EI): *m*/*z* (%): 234 (0.3) [M^+], 105 (100), 77 (27).

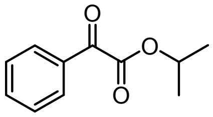

9vγ (CAS No. 31197-66-7)

Isopropyl phenylglyoxylate (9vγ): MS (70 eV, EI): *m*/*z* (%): 192 (0.4) [M^+], 105 (100), 77 (40), 51 (16).

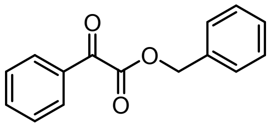

9va (CAS No. 62977-82-6)

Benzyl phenylglyoxylate (9va): MS (70 eV, EI): *m*/*z* (%): 105 (100), 91 (88), 77 (53), 65 (12), 51 (20).

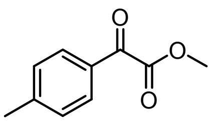

9wi (CAS No. 34966-53-5)

Methyl (4-methylphenyl)glyoxylate (9wi): MS (70 eV, EI): *m*/*z* (%): 178 (2) [M^{+}], 119 (100), 91 (53), 65 (21).

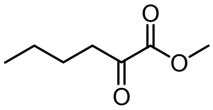

9xi (CAS No. 6395-83-1)

Methyl 2-oxohexanoate (9xi): MS (70 eV, EI): *m*/*z* (%): 144 (8) [M^{+}], 85 (84), 57 (100), 55 (10).

Table S1 Solvent effect[a]

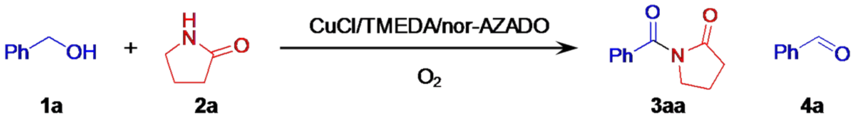

Entry	Solvent	Conv. of **1a** (%)	Yield (%)	
			**3aa**	**4a**
1	THF	>99	>99	<1
2	DCM	>99	13	78
3	MeCN	84	1	72

[a]Reaction conditions: **1a** (0.5 mmol), **2a** (0.55 mmol), CuCl (5 mol%), TMEDA (5 mol%), nor-AZADO (3 mol%), MS 3A (200 mg), solvent (2.5 mL), O_2 (balloon), room temp. (ca. 23°C), 5 min. Conversions and yields were determined by GC analysis.

Table S2 Optimization of the reaction conditions for the reaction of benzyl alcohol (**1a**) and benzamide (**2i**)[a]

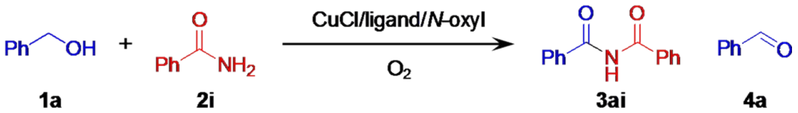

Entry	Ligand	*N*-Oxyl	Base	Conv. of **1a** (%)	Yield (%)	
					**3ai**	**4a**
1[b]	TMEDA	nor-AZADO	none	>99	12	74
2[c]	TMEDA	nor-AZADO	none	>99	24	73
3[d]	TMEDA	nor-AZADO	none	>99	84	12
4[c,e]	TMEDA	nor-AZADO	none	83	<1	70
**5**[c]	**TMEDA**	**nor-AZADO**	***t*BuOK**	**>99**	**>99**	**<1**
6	bpy	nor-AZADO	*t*BuOK	>99	13	81
7	4Mebpy	nor-AZADO	*t*BuOK	82	1	63
8	6Mebpy	nor-AZADO	*t*BuOK	85	1	77
9	4MeObpy	nor-AZADO	*t*BuOK	>99	51	42
10	phen	nor-AZADO	*t*BuOK	>99	44	47
11	MeOphen	nor-AZADO	*t*BuOK	46	<1	33
**12**	**TMEDA**	**nor-AZADO**	***t*BuOK**	**>99**	**72**	**25**
**13**[f]	**TMEDA**	**nor-AZADO**	***t*BuOK**	**>99**	**>99**	**<1**
14	TEEDA	nor-AZADO	*t*BuOK	>99	60	35
15	TMEDA	TEMPO	*t*BuOK	43	<1	30
16	TMEDA	ABNO	*t*BuOK	>99	26	61
17	TMEDA	keto-ABNO	*t*BuOK	34	<1	24
18	TMEDA	AZADO	*t*BuOK	>99	69	24

19	TMEDA	1-Me-AZADO	^{t}BuOK	>99	29	46
20	TMEDA	5-F-AZADO	^{t}BuOK	>99	67	26
21	TMEDA	4-Cl-AZADO	^{t}BuOK	>99	16	64
22	TMEDA	nor-AZADO	DBU	>99	10	77
23	TMEDA	nor-AZADO	DMAP	>99	5	80
24	TMEDA	nor-AZADO	K_2CO_3	>99	13	74

[a]Reaction conditions for entries 1–5: **1a** (0.5 mmol), **2i** (0.55 mmol), CuCl, TMEDA (1 eq.), nor-AZADO (5 mol%), MS 3A (200 mg), THF (2.5 mL), O_2 (balloon), room temp. (ca. 23°C), 1 h; for 6–24: **1a** (0.5 mmol), **2i** (0.55 mmol), CuCl (5 mol%), ligand (5 mol%), *N*-oxyl (3 mol%), base (1 eq.), MS 3A (200 mg), THF (2.5 mL), O_2 (balloon), room temp. (ca. 23°C), 1 h. Conversions and yields were determined by GC and HPLC analysis. [b]CuCl (10 mol%). [c]CuCl (20 mol%), [d]CuCl (100 mol%). [e]**3ai** (20 mol%), half scale. [f]4 h.

Table S3 Control experiments for the reaction of benzaldehyde (**4a**) and 2-pyrrolidone (**2a**)[a]

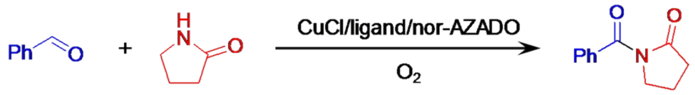

4a **2a** **3aa**

Entry	Cu source	Ligand	*N*-Oxyl	Time (min)	Conv. of **4a** (%)	Yield of **3aa** (%)
**1**	**CuCl**	**TMEDA**	**nor-AZADO**	**2.5**	**98**	**98**
2	CuCl	bpy	nor-AZADO	2.5	25	17
**3**	**CuCl**	**TMEDA**	**nor-AZADO**	**5**	**>99**	**>99**
4	–	TMEDA	nor-AZADO	5	21	<1
5	CuCl	–	nor-AZADO	5	19	<1
6	CuCl	TMEDA	–	5	24	<1
7[b]	CuCl	TMEDA	nor-AZADO	5	24	6
8[c]	CuCl	TMEDA	nor-AZADO	5	7	1

[a]Reaction conditions: **1a** (0.5 mmol), **2a** (0.55 mmol), CuCl (5 mol%), ligand (5 mol%), nor-AZADO (3 mol%), MS 3A (200 mg), THF (2.5 mL), O_2 (balloon), room temp. (ca. 23°C). Conversion and yields were determined by GC analysis. [b]Without MS 3A. [c]Ar (1 atm).

Table S4 Effect of copper sources for the reaction of benzaldehyde (**4a**) and benzamide (**2i**) in the presence of ^{t}BuOK[a]

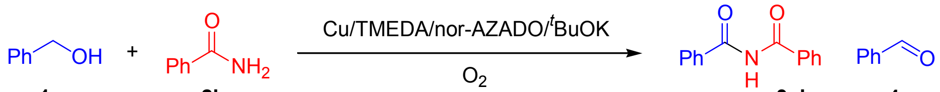

Entry	Cu source	Time (h)	Conv. of	Yield (%)	
			**1a** (%)	**3ai**	**4a**
1	CuCl	1	>99	72	25
2	CuCl	4	>99	>99	<1
3	CuBr	1	>99	75	23
4	CuBr	4	>99	>99	<1
5	CuI	1	>99	66	27
6	CuI	4	>99	>99	<1
7	CuOAc	1	>99	69	28
8	CuOAc	4	>99	>99	<1
9	$CuCl_2$	1	>99	74	22
10	$CuCl_2$	4	>99	>99	<1

[a]Reaction conditions: **1a** (0.5 mmol), **2i** (0.55 mmol), Cu source (5 mol%), TMEDA (5 mol%), nor-AZADO (3 mol%), ^{t}BuOK (1 eq.), MS 3A (200 mg), THF (2.5 mL), O_2 (balloon), room temp. (ca. 23°C). Conversion and yields were determined by GC and HPLC analysis.

Table S5 Control experiments for the reaction of benzaldehyde (**4a**) and benzamide (**2i**)[a]

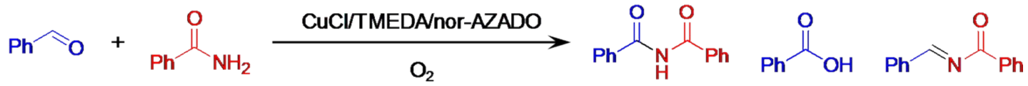

Entry	Cu source	Ligand	*N*-Oxyl	Conv. of	Yield (%)		
				**4a** (%)	**3ai**	**5a**	**6ai**
1	CuCl	TMEDA	nor-AZADO	69	54	<1	14
2	–	TMEDA	nor-AZADO	10	<1	<1	<1
3	CuCl	–	nor-AZADO	42	28	<1	7
4	CuCl	TMEDA	–	34	<1	<1	24
5[b]	CuCl	TMEDA	nor-AZADO	34	14	7	<1
6[c]	CuCl	TMEDA	nor-AZADO	13	2	<1	<1

[a]Reaction conditions: **4a** (0.5 mmol), **2i** (0.55 mmol), CuCl (5 mol%), TMEDA (5 mol%), nor-AZADO (3 mol%), ^{t}BuOK (1 eq.), MS 3A (200 mg), THF (2.5 mL), O_2 (balloon), room temp. (ca. 23°C), 1 h. Conversion and yields were determined by GC and HPLC analysis. [b]Without MS 3A. [c]Ar (1 atm).

Alcohols (**1**)

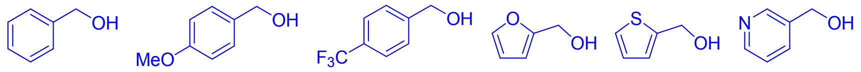

1a **1b** **1c** **1d** **1e** **1f**

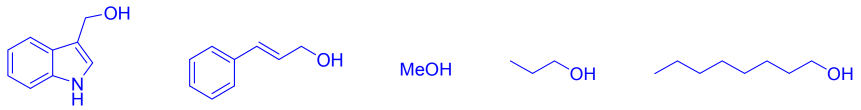

1g **1h** **1i** **1j** **1k**

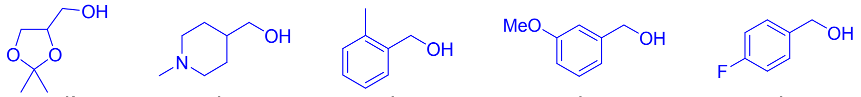

1l **1m** **1n** **1o** **1p**

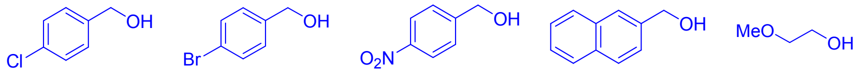

1q **1r** **1s** **1t** **1u**

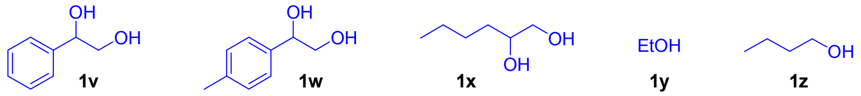

1v **1w** **1x** **1y** **1z**

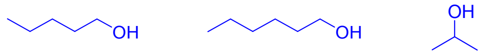

1α **1β** **1γ**

Amides (**2**)

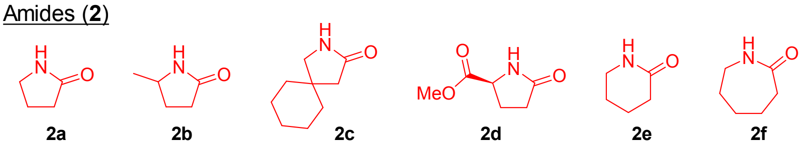

2a **2b** **2c** **2d** **2e** **2f**

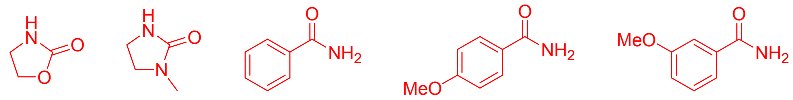

2g **2h** **2i** **2j** **2k**

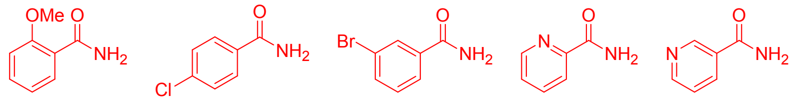

2l **2m** **2n** **2o** **2p**

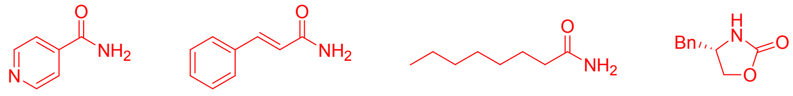

2q **2r** **2s** **2t**

Amines (**7**)

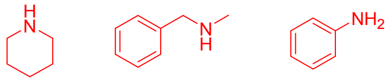

7a **7b** **7c**

Fig. S1 Substrates used in this study.

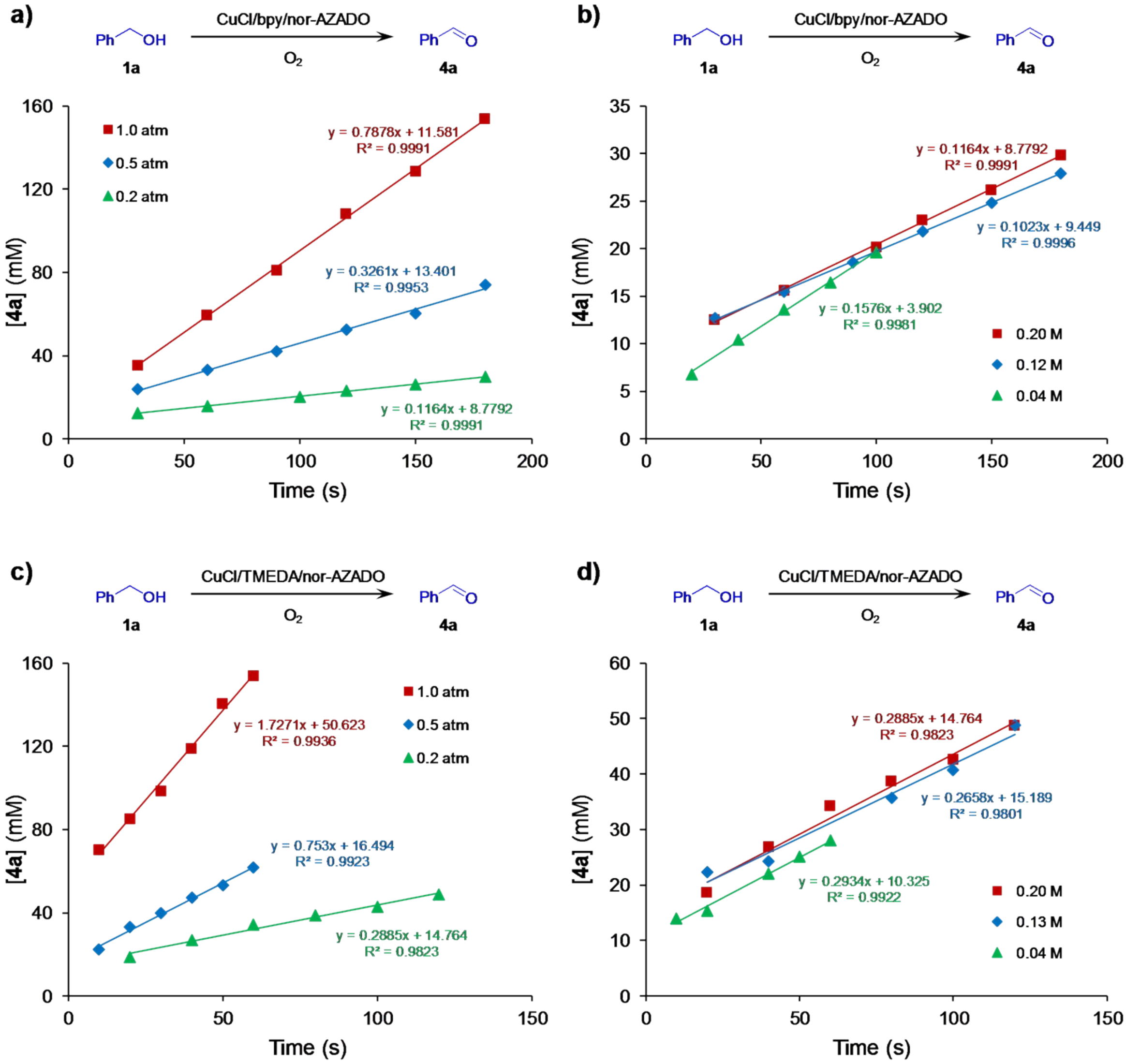

Fig. S2 Reaction profiles for a) the yield of **4a** with different partial pressure of O_2 in the presence of CuCl/bpy/nor-AZADO catalyst, b) the yield of **4a** with different initial concentrations of **1a** in the presence of CuCl/bpy/nor-AZADO catalyst, c) the yield of **4a** with different partial pressure of O_2 in the presence of CuCl/TMEDA/nor-AZADO catalyst, and d) the yield of **4a** with different initial concentrations of **1a** in the presence of CuCl/TMEDA/nor-AZADO catalyst. Reaction conditions for a): **1a** (0.2 M), CuCl (10 mM), bpy (10 mM), nor-AZADO (6 mM), MS 3A (200 mg), THF (2.5 mL), O_2 (0.2–1.0 atm), room temp. (ca. 23°C); for b): **1a** (0.04–0.20 M), CuCl (10 mM), bpy (10 mM), nor-AZADO (6 mM), MS 3A (200 mg), THF (2.5 mL), O_2 (0.2 atm), room temp. (ca. 23°C); for c): **1a** (0.2 M), CuCl (10 mM), TMEDA (10 mM), nor-AZADO (6 mM), MS 3A (200 mg), THF (2.5 mL), O_2 (0.2–1.0 atm), room temp. (ca. 23°C); for d): **1a** (0.04–0.20 M), CuCl (10 mM), TMEDA (10 mM), nor-AZADO (6 mM), MS 3A (200 mg), THF (2.5 mL), O_2 (0.2 atm), room temp. (ca. 23°C). Yields were determined by GC analysis.

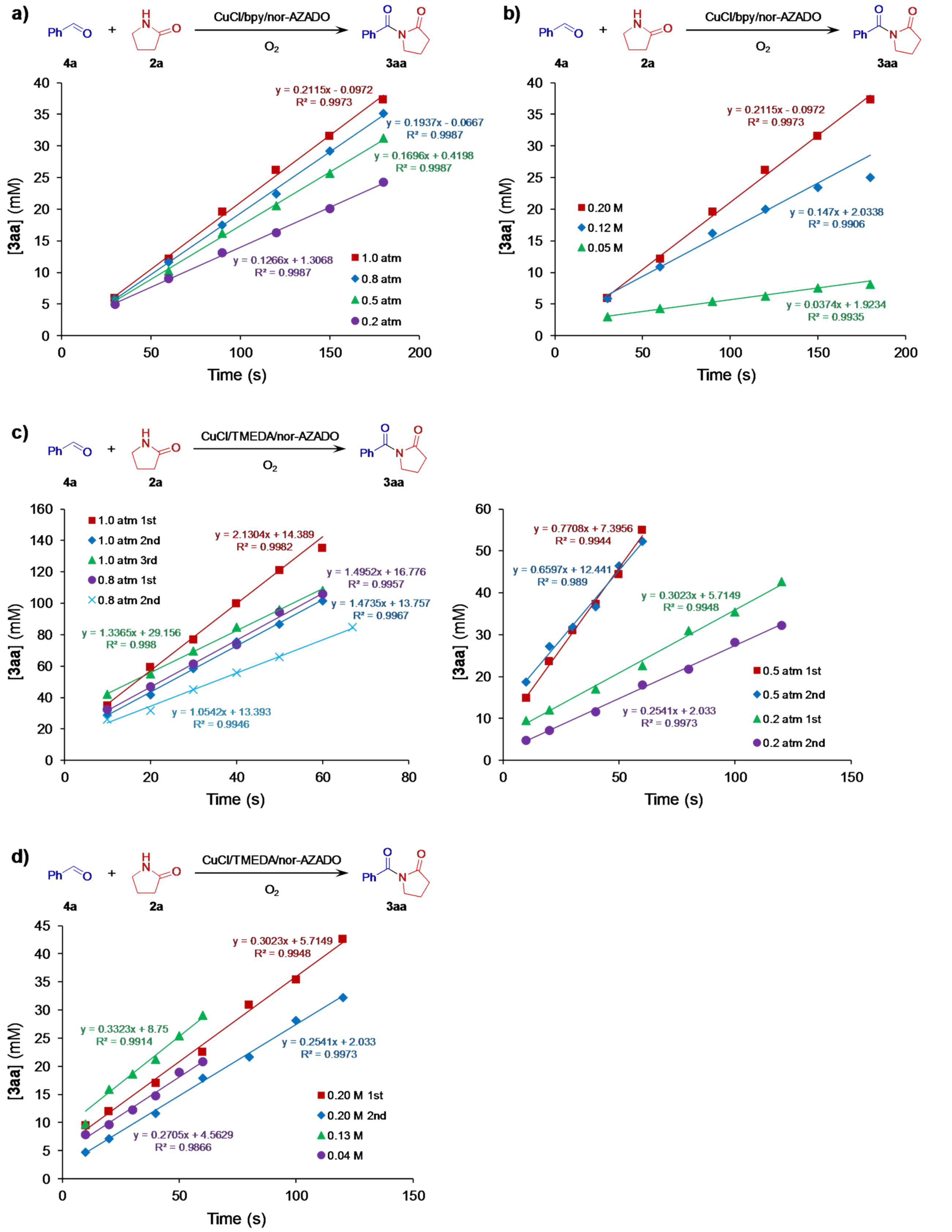

Fig. S3 Reaction profiles for a) the yield of **3aa** with different partial pressure of O_2 in the presence of CuCl/bpy/nor-AZADO catalyst, b) the yield of **3aa** with different initial concentrations of **4a** in the presence

of CuCl/bpy/nor-AZADO catalyst, c) the yield of **3aa** with different partial pressure of O_2 in the presence of CuCl/TMEDA/nor-AZADO catalyst, and d) the yield of **3aa** with different initial concentrations of **4a** in the presence of CuCl/TMEDA/nor-AZADO catalyst. Reaction conditions for a): **4a** (0.20 M), **2a** (0.22 M), CuCl (10 mM), bpy (10 mM), nor-AZADO (6 mM), MS 3A (200 mg), THF (2.5 mL), O_2 (0.1–1.0 atm), room temp. (ca. 23°C); for b): **4a** (0.05–0.20 M), **2a** (0.22 M), CuCl (10 mM), bpy (10 mM), nor-AZADO (6 mM), MS 3A (200 mg), THF (2.5 mL), O_2 (1.0 atm), room temp. (ca. 23°C); for c): **4a** (0.20 M), **2a** (0.22 M), CuCl (10 mM), bpy (10 mM), nor-AZADO (6 mM), MS 3A (200 mg), THF (2.5 mL), O_2 (0.1–1.0 atm), room temp. (ca. 23°C); for d): **4a** (0.04–0.20 M), **2a** (0.22 M), CuCl (10 mM), bpy (10 mM), nor-AZADO (6 mM), MS 3A (200 mg), THF (2.5 mL), O_2 (0.2 atm), room temp. (ca. 23°C). Yields were determined by GC analysis.
